# The neutralizing antibody titer correlate of COVID-19 risk in the COVID-19 variant immunologic landscape (COVAIL) trial was not modified by SARS-CoV-2 amino acid sequence distances

**DOI:** 10.1016/j.vaccine.2026.128348

**Published:** 2026-02-15

**Authors:** Fei Heng, Craig A. Magaret, Nadine G. Rouphael, Angela R. Branche, Youyi Fong, Lindsay N. Carpp, Chenchen Yu, Shiyu Chen, Bo Zhang, David J. Diemert, Ann R. Falsey, Daniel S. Graciaa, Lindsey R. Baden, Sharon E. Frey, Jennifer A. Whitaker, Susan J. Little, Satoshi Kamidani, Emmanuel B. Walter, Richard M. Novak, Richard Rupp, Lisa A. Jackson, Tara M. Babu, Angelica C. Kottkamp, Anne F. Luetkemeyer, Lilly C. Immergluck, Rachel M. Presti, Martín Bäcker, Patricia L. Winokur, Siham M. Mahgoub, Paul A. Goepfert, Dahlene N. Fusco, Robert L. Atmar, Christine M. Posavad, Jinjian Mu, Mat Makowski, Mamodikoe K. Makhene, Seema U. Nayak, Viviana Simon, Harm van Bakel, Paul C. Roberts, Peter B. Gilbert, Coronavirus Variant Immunologic Landscape Trial (COVAIL) Study Team

**Affiliations:** a Department of Mathematics and Statistics, University of North Florida, Jacksonville, FL, USA; b Vaccine and Infectious Disease Division, Fred Hutchinson Cancer Center, Seattle, WA, USA; c Hope Clinic, Emory University, Decatur, GA, USA; d Vaccine and Treatment Evaluation Unit, University of Rochester, Rochester, NY, USA; e Public Health Sciences Division, Fred Hutchinson Cancer Center, Seattle, WA, USA; f Department of Biostatistics, School of Public Health, University of Washington, Seattle, WA, USA; g George Washington Vaccine Research Unit, George Washington University, Washington, DC, USA; h Vaccine and Treatment Evaluation Unit, University of Rochester, Rochester, NY, USA; i Department of Medicine, Brigham and Women’s Hospital, Harvard Medical School, Boston, MA, USA; j Center for Vaccine Development, Saint Louis University, MO, USA; k Department of Molecular Virology and Microbiology and Department of Medicine, Baylor College of Medicine, Houston, TX, USA; l Division of Infectious Diseases and Global Public Health, Department of Medicine, University of California, La Jolla, San Diego, CA, USA; m Center for Childhood Infections and Vaccines, Children’s Healthcare of Atlanta, GA, USA; n Department of Pediatrics, Emory University, Atlanta, GA, USA; o Duke Human Vaccine Institute, Duke University School of Medicine, Durham, NC, USA; p Project WISH, University of Illinois at Chicago, Chicago, IL, USA; q Department of Pediatrics, University of Texas Medical Branch, Galveston, TX, USA; r Kaiser Permanente Washington Health Research Institute, Seattle, WA, USA; s Division of Allergy and Infectious Diseases, Department of Medicine, University of Washington, Seattle, WA, USA; t Vaccine and Treatment Evaluation Unit, Manhattan Research Clinic, New York University Grossman School of Medicine, New York, NY, USA; u Division of HIV, Infectious Diseases and Global Medicine, Zuckerberg San Francisco General Hospital, University of California, San Francisco, CA, USA; v Clinical Research Center, Department of Microbiology, Biochemistry, and Immunology, Morehouse School of Medicine, Atlanta, GA, USA; w Department of Medicine, Washington University School of Medicine, St Louis, MO, USA; x Vaccine and Treatment Evaluation Unit, Long Island Research Clinic, New York University, Long Island School of Medicine, Mineola, NY, USA; y Department of Medicine, University of Iowa College of Medicine, Iowa City, IA, USA; z Howard University College of Medicine, Howard University Hospital, Washington, DC, USA; aa Department of Medicine, University of Alabama at Birmingham, AL, USA; ab Department of Medicine, Tulane University School of Medicine, New Orleans, LA, USA; ac Infectious Diseases Clinical Research Consortium (IDCRC) Laboratory Operations Unit, Fred Hutchinson Cancer Center, Seattle, WA, USA; ad Department of Laboratory Medicine and Pathology, University of Washington, Seattle, WA, USA; ae The Emmes Company, LLC, Rockville, MD, USA; af Division of Microbiology and Infectious Diseases, National Institute of Allergy and Infectious Diseases, National Institutes of Health, Bethesda, MD, USA; ag Department of Microbiology, Icahn School of Medicine at Mount Sinai, New York, NY, USA; ah Center for Vaccine Research and Pandemic Preparedness (C-VaRPP), Icahn School of Medicine at Mount Sinai, New York, NY, USA; ai Department of Pathology, Molecular, and Cell-Based Medicine, Icahn School of Medicine at Mount Sinai, New York, NY, USA; aj Division of Infectious Diseases, Department of Medicine, Icahn School of Medicine at Mount Sinai, New York, NY, USA; ak The Global Health and Emerging Pathogens Institute, Icahn School of Medicine at Mount Sinai, New York, NY, USA; al Department of Genetics and Genomic Sciences, Icahn School of Medicine at Mount Sinai, New York, NY, USA.; am Department of Artificial Intelligence and Human Health, Icahn School of Medicine at Mount Sinai, New York, NY, USA; an Icahn Genomics Institute, Icahn School of Medicine at Mount Sinai, New York, NY, USA

**Keywords:** Deep mutational scanning, Immune correlate of protection, mRNA vaccine, Neutralizing antibody escape, Randomized clinical trial, Recombinant protein vaccine

## Abstract

In the Coronavirus Variant Immunologic Landscape Trial (COVAIL) conducted in the United States in 2022–2023, 985 participants received a second COVID-19 booster with one of twelve monovalent or bivalent mRNA inserts. Pseudovirus serum inhibitory dilution 50% neutralizing antibody titer (nAb titer) measured two-weeks post booster significantly associated with lower COVID-19 incidence over six months follow-up in this trial. COVAIL investigators sequenced SARS-CoV-2 Spike amino acid sequences for all COVID-19 cases, with a sequence successfully obtained from 129 of 195 cases. For COVID-19 endpoint cases we calculated five distances of the case-causing sequence to a reference sequence, the first two physico-chemical weighted Hamming distances of Spike or receptor binding domain (RBD) to a participant’s nearest Spike or RBD vaccine-insert sequence, and the other three estimated degrees of neutralizing antibody escape from the XBB.1.5 RBD strain calculated with deep mutational scanning. Hypothesizing that the nAb titer correlate of risk may have a stronger association with COVID-19 when focusing on COVID-19 infections more closely matched to the vaccine insert in Spike or RBD amino acid sequence or with lower RBD antibody escape score, we tested this hypothesis for the combined group receiving a monovalent Prototype (ancestral strain) booster (*n* = 143) and for the combined group receiving an Omicron-containing booster (*n* = 744). For both combined groups, the nAb titer correlate of risk did not significantly vary across any of the assessed sequence distances from the vaccine insert (all *p*-values >0.10), although RBD Hamming distance had point estimates consistent with a weakening correlate with distance, motivating further exploration in settings with greater antigenic heterogeneity. Indeed, statistical power was bounded by the limited antigenic variability of viruses infecting trial participants over the follow-up period (April 21, 2022 to May 25, 2023), which spanned only a 3.02-fold nAb titer range of differential sensitivity to sera from XBB.1.5-infected individuals. ClinicalTrials.gov Identifier: NCT05289037.

## 1. Introduction

COVID-19 vaccines based on the ancestral (GenBank accession no. MN908947) strain have been widely phased out and replaced by ones adapted [1–4] to counteract decreased vaccine effectiveness [5–10] against COVID-19 caused by emerging SARS-CoV-2 variants [11,12]. Booster vaccine doses [Prototype or variant-adapted] were also introduced, demonstrating efficacy especially over short-term follow up [13–22]. The FDA recommended a BA.4/BA.5 strain booster in fall of 2022 and an updated XBB.1.5 strain booster in fall of 2023. In subsequent updates, strain selection has continued to evolve based on global surveillance, with the 2024–2025 season incorporating variants from the XBB lineage and more recently, JN.1 sublineages.

In this variant boost era, the randomized, open-label COVID-19 Variant Immunologic Landscape (COVAIL) vaccine trial was conducted to assess the safety and immunogenicity of variant COVID-19 boosters. These boosters elicited cross-reactive neutralizing antibodies against multiple variants — D614G, Beta, Delta, BA.1, and BA.4/BA.5 — but with antibody waning over three months and markedly reduced titers against newer Omicron subvariants (e.g., BQ.1.1 and XBB.1) [23,24].

Neutralizing antibody titer to the ancestral or D614G strain was established as a correlate of protection (CoP) [25,26] against COVID-19 caused by pre-Delta strains [27] and has been used as a surrogate endpoint to guide regulatory decisions [28–32]. Two analyses of COVAIL data investigated performance of pseudovirus inhibitory serum dilution 50% neutralizing antibody titer (nAb titer) as an immune correlate in the Omicron-variant era [33,34]. Fong et al., focusing on Stage 3 with recombinant protein vaccines, reported that peak nAb titer [Day 15 (D15) post-second boost] was an inverse correlate of risk (CoR) of BA.4/BA.5 COVID-19 over ~6-months post-D15 [33]. Although BA.4/BA.5 dominated during follow-up, the results did not support that matching the variant titer to the circulating strain was needed to preserve the correlate [33]. Zhang et al. reported similar results for recipients of a one-dose mRNA second vaccine boost in Stages 1, 2, and 4, where again matching the variant titer to BA.4/BA.5 did not yield a stronger CoR of BA.4/BA.5 COVID-19 as compared to the D614G titer [34], although it did improve statistical precision.

The Fong et al. and Zhang et al. analyses investigated the nAb titer as a correlate of overall or lineage-specific COVID-19. Additional insights into immune correlates can be potentially gained by accounting for pathogen sequence features beyond lineage. For example, based on pathogen sequences obtained from participants experiencing a given infectious disease endpoint, sieve analyses [35] assess how vaccine efficacy varies with pathogen sequence features, as was done for the Ad26. COV2⋅S [36] and mRNA-1273 [37] COVID-19 vaccines and for other vaccines and pathogens (e.g., refs. [38–41]). In a further step, pathogen sequence data may be incorporated into immune correlates analyses, for example by assessing nAb titer as a correlate of a disease endpoint specific to a particular genotypic or immunotypic distance of the pathogen to a vaccine-insert sequence. Sun et al. performed such an analysis of data from a randomized, placebo-controlled HIV-1 vaccine efficacy trial, finding that the inverse correlation of homologous anti-Env V2 IgG binding antibody concentration with acquisition of HIV-1 infection was stronger for viruses with shorter V2 amino acid sequence distances to the vaccine strains [42]. Qi et al. performed a similar analysis of a tetravalent dengue vaccine efficacy trial, finding that the inverse correlation of homologous average nAb titer (against the four vaccine strains) with symptomatic, virologically-confirmed dengue was stronger for dengue viruses with shorter amino acid sequence distances (defined at nAb contact sites) to the vaccine strains [43]. Such host-pathogen integrated analyses can demonstrate immunological relevance of sequence distance and may inform development of surrogate endpoints. The use of such integrative methods in COVID-19 has been limited to date, and their potential to inform immunobridging strategies [28,32] for approving refined or new vaccines remains underutilized. This study offers a critical opportunity to assess their applicability under real-world variant dynamics.

The question we address is whether the nAb titer correlate of risk becomes weaker against sequence-specific COVID-19 with greater distance from the vaccine-insert sequence or with greater estimated antibody escape score distance from XBB.1.5 based on deep mutational scanning experiments of XBB.1.5 using sera from XBB.1.5-infected individuals. If the answer is affirmative for a given sequence distance, then it suggests that the distance is immunologically relevant and adds information to the nAb titer immune correlate beyond the measurement of the titer alone. Our objective is to assess D15 nAb titer against the vaccine strain as a correlate of risk (CoR) of sequence-distance-specific COVID-19 over 6 months of follow-up post booster, the same period of follow-up studied previously [33,34].

## 2. Methods

### 2.1. Background on COVAIL

COVAIL was conducted in four sequential stages: Stage 1 began in March 2022 and Stage 4 in October 2022. The study enrolled adults in the United States stratified by age (18–64, 65+) and prior SARS-CoV-2 infection history, deemed to be in stable health, and previously vaccinated as defined by confirmed receipt of a complete primary and booster COVID-19 vaccine series, either homologous or heterologous, with an FDA authorized/approved vaccine at least 16 weeks prior to study vaccine dose 1 [23,24]. Participants were randomized to one of a stage-specific selection of monovalent or bivalent COVID-19 vaccine second boosters [mRNA or adjuvanted recombinant protein; Prototype, Beta (B.1.351), Delta (B.1.617.2), Omicron BA.1 (B.1.1.529.1), or Omicron BA.4/BA.5 (B.1.1.529.4/B.1.1.529.5) Spike inserts] [23,24]. See [23] for complete inclusion/exclusion criteria.

### 2.2. Two vaccine groups for analyses

We assessed our objective for each of two groups of one-dose mRNA vaccine arms: vaccines with an Omicron insert (monovalent or bivalent), and vaccines with a monovalent Prototype insert, referred to as the Omicron Vaccine Group and the Prototype Vaccine Group. The former group comprised arms 2, 4–6, 8–9, 12, 16, 17 whereas the latter group comprised arms 1 and 7 (Table 1 and refs. [23, 34]). The deep mutational scanning distances were studied for the Omicron Vaccine Group alone given that all infecting viruses were with Omicron strains and the deep mutational scanning experiments measured antibody escape from the XBB.1.5 Omicron reference strain [44].

### 2.3. Definitions of naïve and non-naive

As in Zhang et al. [34], SARS-CoV-2 non-naïve (“Non-naïve”) was defined as having a self-reported history of prior SARS-CoV-2 infection or having detectable anti-N antibodies [defined as Elecsys Anti-SARS-CoV-2 assay (Roche) cutoff index ≥1.0] at D1. Otherwise, a participant was considered SARS-CoV-2 naïve (“Naïve”).

### 2.4. Definition of COVID-19 endpoint and time frame for occurrence

As in [34], the COVID-19 endpoint was a self-reported positive SARS-CoV-2 test (RT-PCR or antigen test) or study-conducted positive SARS-CoV-2 test (nasal swab and subsequent nucleic acid amplification test at an unscheduled illness visit) with onset date the earliest positive test date. COVID-19 endpoints for correlates assessment occurred in the time period 7 to 188 days post-D15, with evaluable cases defined as COVID-19 endpoints in participants in the correlates analysis per-protocol cohort (defined in [34]) during this time period. Non-cases were participants in the correlates analysis per-protocol cohort with no evidence of SARS-CoV-2 infection during 7 to 188 days post-D15.

### 2.5. Viral genome sequencing

Viral RNA was isolated from nasopharyngeal swabs suspended in PBS or viral transport medium (VTM) using the Chemagic^™^ Viral DNA/RNA 300 Kit H96 (PerkinElmer, CMG-1033-S) on a Chemagic^™^ 360 automated extraction platform, following the manufacturer’s instructions. SARS-CoV-2 RNA was quantified by real-time RT-PCR targeting the N1 region using the CDC/NCIRD/DVD 2019-nCoV assay (IDT 2019-nCoV RUO Kit, 10006713). Specimens with cycle threshold (Ct) values ≤32 were selected for downstream sequencing analyses. Complementary DNA synthesis and whole-genome amplification were carried out using two custom primer pools designed to generate overlapping 1.5-kb and 2-kb amplicons spanning the viral genome, as previously reported [45]. Paired-end (2×150bp) Nextera XT libraries (Illumina, cat. FC-131–1096) were prepared from amplicons and sequenced on a MiSeq instrument. Consensus genomes were generated using the Virus Reference-based Assembly Pipeline and IDentification (vRAPID) package [46]. Genomes with >95% coverage were genotyped with Nextclade CLI (v2.13.0 & v2.14.0) partially Aliased assignments [47] and pangolin (v4.1.3 & v4.3) [48].

### 2.6. Distances of spike and RBD amino acid sequences to reference sequences

We studied two physico-chemical weighted Hamming distances of a Spike amino acid (AA) sequence to the vaccine-insert sequence(s), computed for whole spike and for RBD. These distances were previously studied in COVID-19 vaccine trial sieve analyses [35,36]. The Prototype Vaccine Group boosters contain the Wuhan-Hu-1 reference sequence (GenBank accession number: MN908947), which is also referred to as Wuhan, Ancestral, or Prototype [49].

For the Omicron Vaccine Group, we also include three antibody escape score distances defined based on deep mutational scanning (DMS). The distances were computed based on antibodies in sera sampled from individuals infected with the Omicron XBB.1.5 SARS-CoV-2 strain in 2022–2023. The XBB.1.5 escape score values, calculated across full spike, were reported in Fig. 4a of Dadonaite et al. [44]. For each spike AA sequence from an Omicron Vaccine Group recipient who acquired a COVID-19 endpoint we calculate an antibody-escape score of the RBD sequence relative to the XBB.1.5 RBD sequence. The final distance used for the analysis is the weighted RBD Hamming distance to the XBB.1.5 RBD reference strain, modified such that if an AA site mutation away from XBB.1.5 matches the residue in Ancestral, then the mutation is removed by assigning the position a score of zero. In addition, if the mutation away from XBB.1.5 matches an individual participant’s vaccine-insert residue (or either vaccine insert for a bivalent vaccine), then it is removed by assigning a score of zero. This distance is motivated by observations from influenza antibody escape [50].

A potential limitation of the DMS-based distances is that many RBD AA positions have small escape scores close to zero, such that including all RBD positions may have more noise compared to distances that only include positions with escape score above a specified threshold. Accordingly, two additional distances are computed, calculated in the same way except only including the 14 RBD sites with greatest escape score from XBB.1.5 in Dadonaite et al. [44] listed in their Fig. 4a: 352, 357, 371, 375, 420, 421, 440, 447, 450, 455, 456, 473, 475, 485, which we refer to as the DMS-escape RBD-3 set of sites. Moreover, for ‘middle ground’ scores between inclusion of all RBD amino acid sites with escape score values (167 sites) and the 14 DMS-escape RBD-3 sites, we plotted the position-specific escape scores across all amino acid sites and sorted them, and searched for a cut-off that majorly reduced the total number of sites but not all the way down to the 14 DMS-escape RBD-3 sites, essentially looking for a kink in the convex hull of the plot. An escape-score cut-off of 0.7 was used to screen-in amino acid sites, which included 48 AA positions on which the DMS-escape RBD-2 distances were computed.

In sum, for the Prototype Vaccine Group, two distances were studied, the spike and RBD weighted Hamming distances to the Ancestral reference sequence, and for the Omicron Vaccine Group, these two distances were also studied using each participant’s Omicron vaccine-insert sequence as the reference sequence. In addition, for the Omicron Vaccine Group the three antibody escape score distances DMS-escape RBD-1, RBD-2, and RBD-3 were studied.

### 2.7. Day 15 neutralizing antibody markers and viral sequence distances analyzed for each of the two groups

For Omicron Vaccine Group vaccine arms, the marker of interest is D15 log10 ID50 to Omicron BA.1, because BA.1 is the vaccine-insert lineage for vaccine arms 2, 4–6, 8–9, 12, 16. For vaccine arm 17, the marker of interest is D15 log10 ID50 to BA.4/BA.5, given the insert lineage is BA.4/BA.5. For Prototype Vaccine Group arms, the marker of interest is D15 log10 ID50 to D614G, because D614G, equal to Wuhan/Ancestral, is the insert strain. The analyses are done pooling Naïve and Non-naïve participants, and supplemental analyses are done only for Naïve participants.

### 2.8. Criteria for whether an analysis is included

Acknowledging that the Sun et al. method [51] only provides worthwhile precision when there is sufficient variability in the mark/distance, each specified data analysis with this method is retained only if there are at least 10 unique mark/distance values represented among evaluable COVID-19 cases. Table 2 lists the analyses that are conducted, where the DMS-escape RBD-2 and RBD-3 analyses are conducted descriptively but not inferentially using Sun et al. because of the 6 and 2 unique values, respectively.

## 3. Statistical methods

### 3.1. Estimation of sequence-distance-specific correlates of risk of COVID-19

The methods of Sun et al. [51] were used to estimate the AA-distance specific hazard ratio (HR) of COVID-19 per unit change in log10 D15 nAb titer (this HR corresponds to the hazard rate of COVID-19 with the given viral AA-sequence distance: the numerator hazard of second booster recipients with a given value of log10 D15 nAb titer, and the denominator hazard of second booster recipients with a log10 D15 nAb titer that is one unit smaller), implemented separately for the Prototype and Omicron Vaccine Groups. The methods estimate distance-specific HRs ranging over the spectrum of distance values using nonparametric kernel smoothing. The analyses right-censor participants at 188 days post D15 or at loss to follow-up if it occurred earlier. Results are presented as point and 95% confidence interval (CI) estimates of distance-specific HRs per unit change in D15 log10 nAb titer over all observed distance values. The methods of Sun et al. [51] were also used to estimate probabilities of distance-specific COVID-19 by 188 days post D15 (ranging over all distances) for subgroups defined by the 10th, 50th, and 90th percentile of the D15 nAb titer.

### 3.2. Hypothesis testing

The methods of Sun et al. [51] were used to test two different hypotheses that distance-specific D15 nAb titer HRs varied with AA distance: The first is that the distance-specific HR departed from unity for at least one distance value (i.e., is nAb titer a CoR for some virus genotypes?), and the second is that the distance-specific HR increased with distance (i.e., does the strength of the CoR become weaker against viruses more distant from the reference virus?). Following Sun et al. the test statistics for evaluating these hypotheses are referred to as T1m and T2m, respectively.

### 3.3. Handling missing viral sequence data

Of the 195 COVID-19 endpoints, 66 (33.8%) are missing viral sequence data, defined by any of the 223 residues in RBD having a missing value. Of the 66 endpoints with missing data, 39 generally did not have sequencing attempted because the cycle threshold value exceeded a threshold for attempting sequencing, and of the 27 partial sequences with missing content, 26 were missing more than 5% of the 223 residues and one was missing 7 residues (3.1%).

Hotdeck (predictive mean matching) multiple imputation is used to accommodate missing sequences, implemented as follows. For each COVID-19 case with a missing genotype, nearest neighborhoods of COVID-19 cases with viral sequence observed are defined based on z-scores of the calendar time of COVID-19 failure time (where the calendar time variable is calculated as the number of days from March 30, 2022). Ten imputed genotypes/marks from the 5-nearest neighborhoods are assigned. The hotdeck multiple imputation procedure is updated compared to that described in Sun et al. [51], to include an ABC bootstrapping of cases that provides proper multiple imputation.

All analyses adjust for baseline risk score and FOI standardized score. Analyses combining over Naïve and Non-naïve participants also adjust for naïve status. The Supplementary Methods provide additional details on the statistical methods, the implementation of the proposed estimation and hypothesis testing procedures, and the choice of bandwidths for kernel smoothing.

## 4. Results

Table 1 summarizes the COVAIL data included in the analyses and reports geometric mean D15 nAb titers for the antigens against which correlates were studied. For the Prototype Vaccine Group, the total sample size was 143 (100 non-cases, 43 cases), with 28 (65.1%) of the cases having SARS-CoV-2 sequence data. For the Omicron Vaccine Group, the total sample size was 744 (592 non-cases, 152 cases), with 101 (66.4%) of the cases having SARS-CoV-2 sequence data. The amount of data was greater for Moderna than Pfizer-BioNTech and for Naïve than Non-naïve. The geometric mean D15 nAb titers were lower in cases than non-cases, reflecting the published correlates results [34].

Table 3 summarizes baseline demographics, focusing on immunity-relevant factors, including stratification by Naïve and Non-naïve. For the Omicron Vaccine Group pooling over Naïve and Non-naïve, 24.9% of participants were age at least 65, 54.7% were female, and the top two vaccination histories were 3 doses of Pfizer-BioNTech mRNA (52.0%) and 3 doses of Moderna mRNA (39.0%), followed by 5–7% each receiving a mismatched mRNA vaccine manufacturer 2-dose primary series and first booster. A greater frequency of Naïve than Non-naïve participants were age at least 65 (34.2% vs. 9.8%) whereas sex and vaccination history had similar distributions between Naïve and Non-naïve. Also for the Omicron Vaccine Group, baseline geometric mean nAb titer against BA.4/BA.5 was 2329 overall, and 1418 and 5180 stratified by Naïve and Non-naïve, respectively, highlighting the immunity advantage of prior infection. Focusing on COVID-19 cases, covariate distributions did not appear different compared to the overall cohorts, except that baseline geometric mean nAb titers were lower in cases for both Naïve (geometric mean 1171) and Non-naïve (2674), which recapitulates the correlate of risk results previously reported [34].

**Fig. 1.**
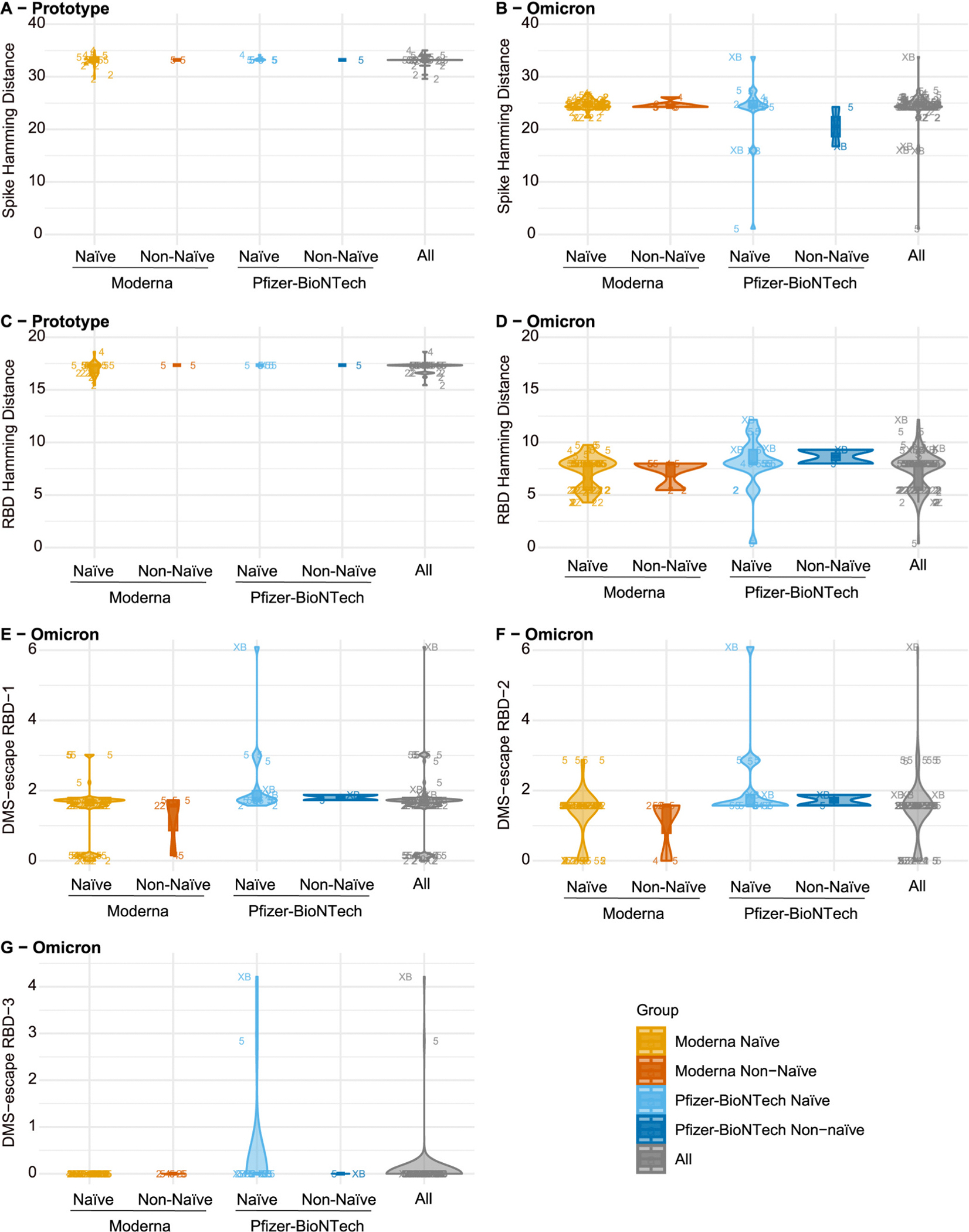
Viral sequence distances of COVID-19 endpoints for the Prototype Vaccine Group and the Omicron Vaccine Group. (A)-(D) show physico-chemical weighted Hamming distances for spike and receptor binding domain (RBD), with (A), (C) for the Prototype Vaccine Group and (B), (D) for the Omicron Vaccine Group. (*E*)-(G) show antibody escape score distances for the Omicron Vaccine Group for deep mutational scanning (DMS)-escape RBD-1, RBD-2, and RBD-3. Information on the COVID-19-causing lineage is represented in the plotting symbol: 2 = BA.2, 4 = BA.4, 5 = BA.5, XB = XBB.1.5, M = lineage information missing.

For each of the five SARS-CoV-2 sequence distances defined in Methods, Fig. 1 shows distributions of the distances of COVID-19 endpoints for the Prototype and Omicron Vaccine Groups, stratified by Moderna/Pfizer-BioNTech booster and Naïve/Non-naïve. The Spike Hamming distances (Panels A, B) had median 33.2 and 24.8 AA mismatches to the closest vaccine strain for the Prototype and Omicron Vaccine Groups, respectively, showing how Omicron-matching moved the vaccine strains closer to the circulating strains. Restricting to the receptor binding domain (RBD), these medians were 17.3 and 8.0 AA mismatches, respectively. The deep mutational scanning (DMS) RBD-1 antibody escape score distances that considered whole RBD (see Methods) had median 1.73 for the Omicron Vaccine Group. A DMS-escape RBD-1 value of 0 represents the XBB.1.5 RBD sequence (no escape) and a value of x = 1.73 can be interpreted as an expected 1.73-fold reduced virus neutralization ID50 titer of sera from convalescent XBB.1.5-strain infected persons against that virus (geometric mean ID50 over individual sera) compared to against the XBB.1.5 reference virus [44]. For the Omicron Vaccine Group, the DMS-escape RBD-2 distances that considered a subset of RBD AA positions had median 1.57, whereas this median was 0 for the DMS-escape RBD-3 distances that further subsetted on the 14 RBD AA positions with greatest escape from XBB.1.5.

Variability of sequence distances across COVID-19 endpoint cases was a driver of statistical power for addressing the posed study question. The Spike Hamming distances were mostly concentrated between 30 and 35 (Prototype Vaccine Group) and between 22 and 27 (Omicron Vaccine Group), whereas the RBD Hamming distances had markedly more heterogeneity for the Omicron Vaccine Group (mostly concentrated between 4 and 12). The range of the DMS-escape RBD-1 distances across Omicron Vaccine Group COVID-19 endpoint cases corresponded to an expected range of nAb titer neutralization sensitivity reduction from no reduction to 3.02-fold reduction compared to the XBB.1.5 strain, and the range of the DMS-escape RBD-2 distances ranged from no reduction to 2.87-fold reduction [these calculations excluded the XB outlier with Hamming distance ~6 for DMS-escape RBD-1 and RBD-2, and ~ 4 for DMS-escape RBD-3 (Fig. 1E–1G)]. All but two of the 101 Omicron Vaccine Group COVID-19 endpoint cases had DMS-escape RBD-3 distance equal to 0, of which 99 were infected with the wildtype (no-escape) XBB.1.5 sequence ARFFDYKGNLFYAG at the 14 high-escape positions, 1 with ARFFDYKGDLFYAG that had XBB.1.5 antibody escape 2.84, and 1 with ARFFDYKGNLLYAG (escape score 4.21) (Table 4). Therefore, almost no circulating strains that caused COVID-19 infections had any of the 14 high-ranked antibody escape mutations from XBB.1.5 while also mismatching the Ancestral strain and the participant’s vaccine sequence(s). This implies insufficient variability to assess how the nAb titer CoR depends on DMS-escape RBD-3 distance, such that these analyses were not conducted; these inferential analyses were also foregone for DMS-escape RBD-2 given its limited variability (see Methods).

Fig. 1 also shows the lineages causing COVID-19 endpoint cases: 7 BA.2, 3 BA.4, and 18 BA.5 cases in the Prototype Vaccine Group, and 27 BA.2, 10 BA.4, 59 BA.5, 1 XZ, 2 XBB.1.1, and 2 XBB.1.5 cases in the Omicron Vaccine Group. Thus 69.8% of all cases were with BA.4 or BA.5 lineages and 26.4% with BA.2. Previously Zhang et al. [34] described the timing of lineage-specific COVID-19 for the original correlates study, with BA.2 dominant in April–May 2022 and BA.4/BA.5 dominant thereafter through the end of follow-up May 25, 2023.

**Fig. 2.**
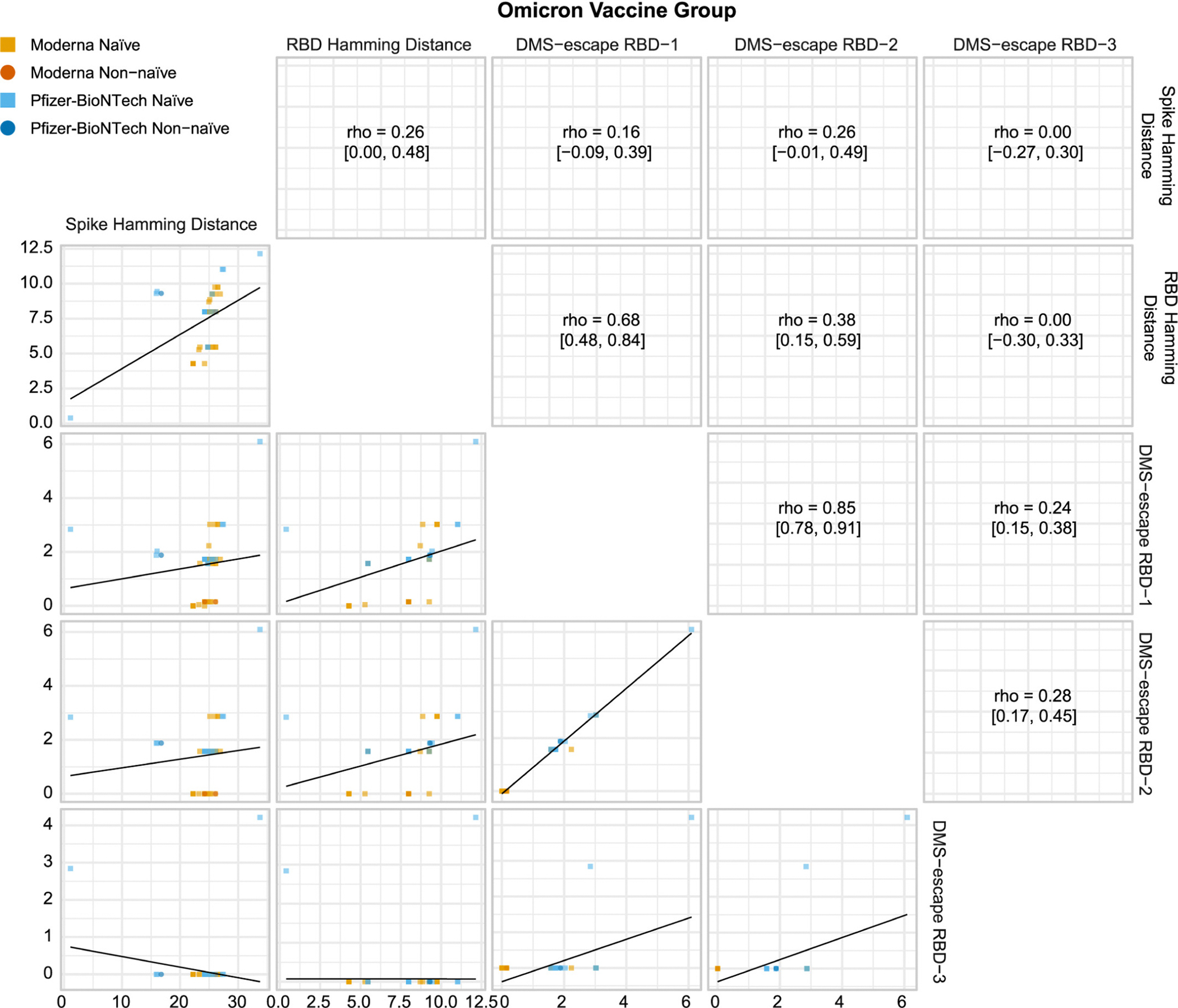
Pairwise correlations of viral sequence distances of COVID-19 endpoints for the Omicron Vaccine Group. Distances are physico-chemical weighted Hamming distances for spike and receptor binding domain (RBD) and the three antibody escape score distances deep mutational scanning (DMS)-escape RBD-1, RBD-2, and RBD-3. Rho, Spearman rank correlation with a 95% confidence interval.

Fig. 2 shows correlations of the viral sequence distances of COVID-19 endpoints for the Omicron Vaccine Group. The Spike and RBD Hamming distances were only moderately correlated [Spearman rank correlation (rho) =0.26]. The Spike Hamming distance was essentially uncorrelated with the three antibody escape score distances (all rho <0.30), whereas the RBD Hamming distance had some correlation with the DMS escape RBD-1 distance (rho = 0.68). The DMS escape RBD-1 and RBD-2 distances were highly correlated (rho = 0.85), which implies that results are expected to be similar for these two distances such that dropping the RBD-2 CoR analyses unlikely missed insights. For the Prototype Vaccine Group, the Spike and RBD weighted Hamming distances were highly correlated (rho = 0.86, Supplementary Fig. 1). The lower correlation of these Hamming distances for the Omicron Vaccine Group was due in part to the heterogeneity of Omicron vaccine-insert sequences (BA.1, BA.4.5).

**Fig. 3.**
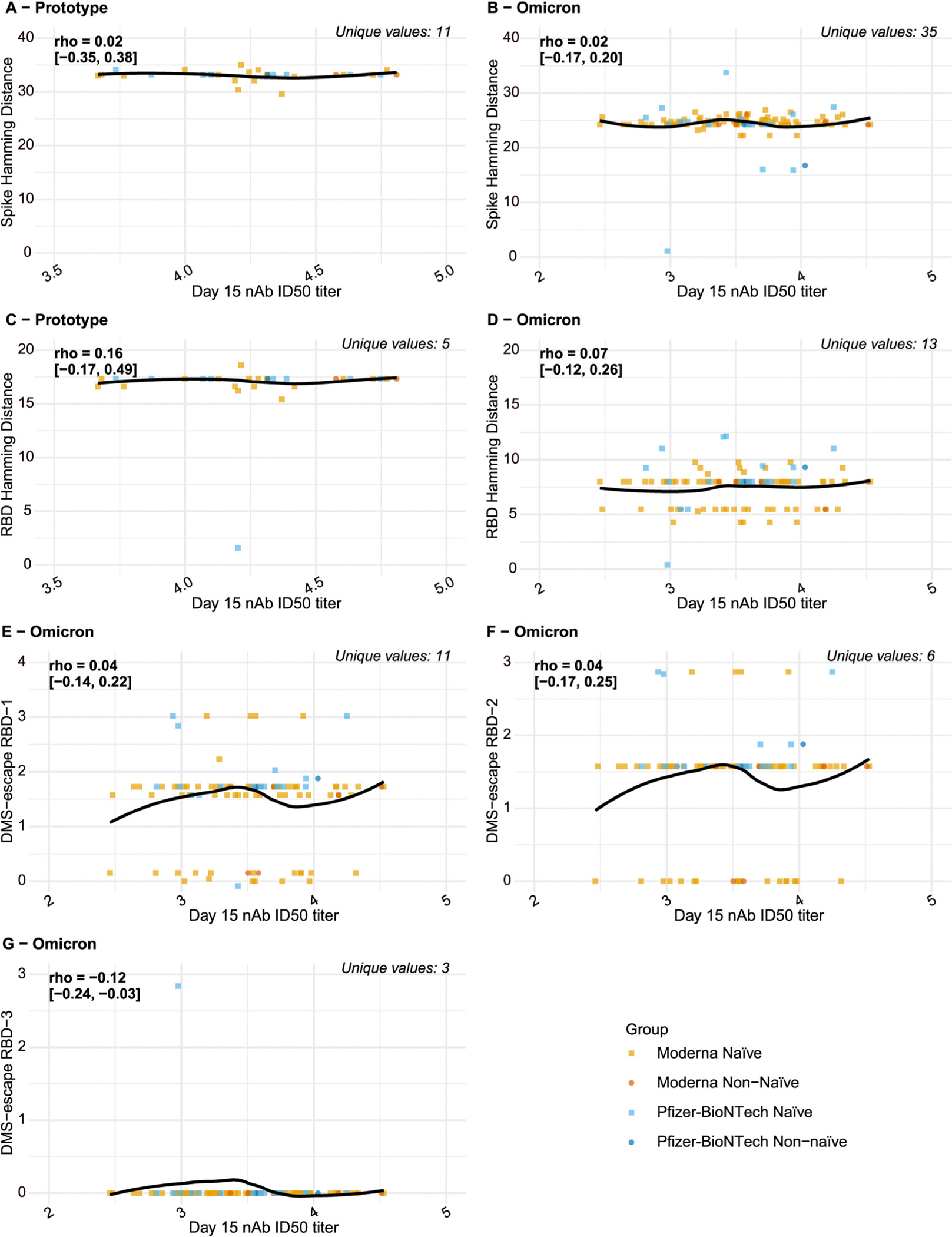
Viral sequence distances vs. log10 D15 nAb titer for COVID-19 endpoint cases for the Prototype Vaccine Group and the Omicron Vaccine Group. For the Prototype Vaccine Group, nAb titer is against D614G. For the Omicron Vaccine Group, nAb titer is against BA.1 except for Arm 17 it is against BA.4/BA.5. Sequence distances are physico-chemical weighted Hamming distances for spike and receptor binding domain (RBD) and 3 antibody escape score distances deep mutational scanning (DMS)-escape RBD-1, RBD-2, RBD-3. Rho, Spearman rank correlation with a 95% confidence interval.

For the viral sequence distances with sufficient variability across COVID-19 endpoint cases to support assessment of distance-specific CoRs, Fig. 3 shows the viral distances versus the D15 nAb titers for the Prototype and Omicron Vaccine Groups, pooling Naïve and Non-naïve participants (Supplemental Fig. 2 restricts to the Naïve cohort). Our targeted hypothesis of the CoR weakening with viral distance would garner some support from a result with higher ID50 titer associated with larger viral sequence distance. However, the Spearman rank correlations were all near zero (all estimates with absolute value ≤0.12 and with six of the seven 95% confidence intervals including zero).

**Fig. 4.**
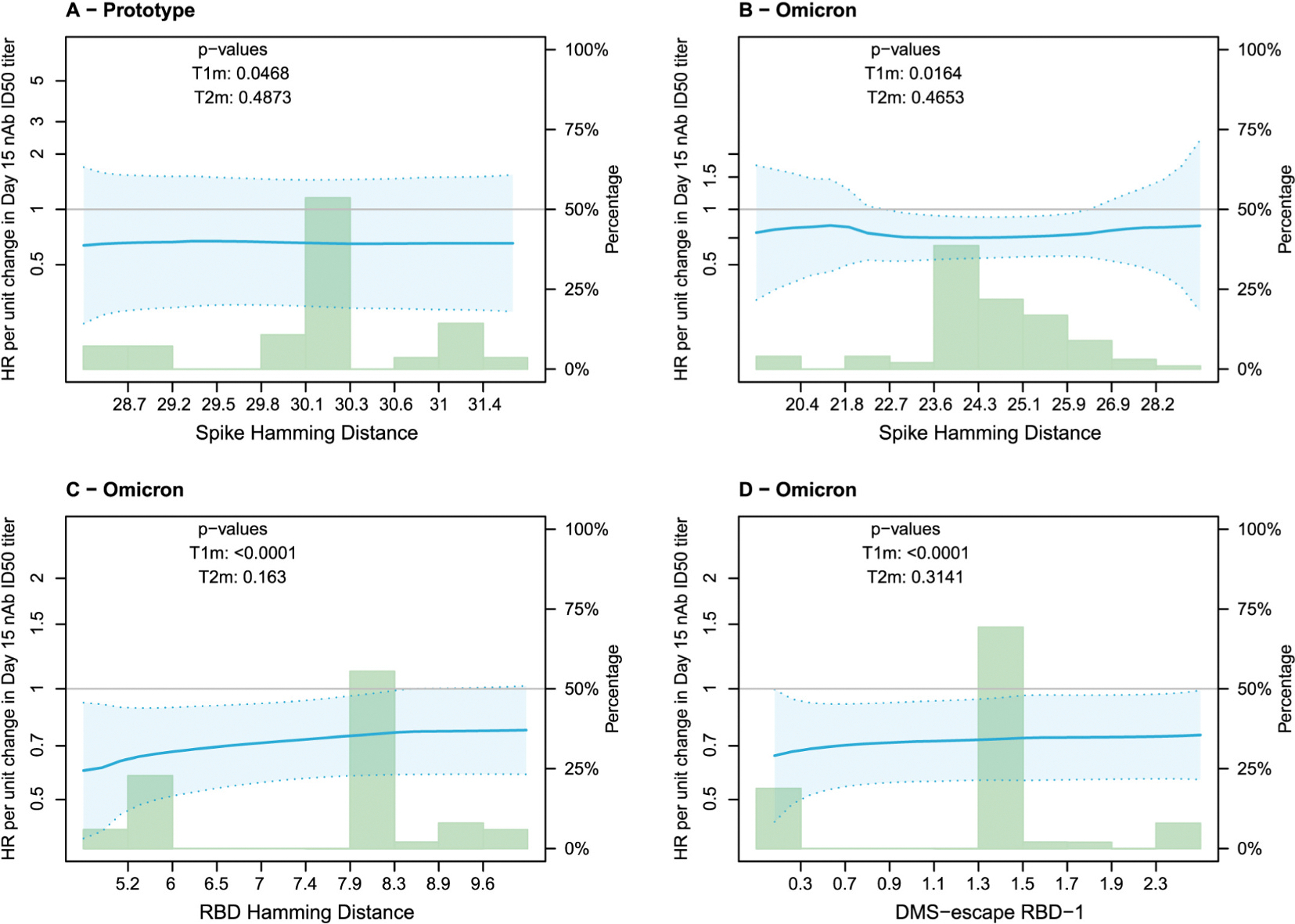
Hazard ratios of viral sequence distance-specific COVID-19 for D15 nAb titer markers for the Prototype and Omicron Vaccine Groups. For the Prototype Vaccine Group, nAb titer is against D614G. For the Omicron Vaccine Group, nAb titer is against BA.1 except for Arm 17 it is against BA.4/BA.5. The 2-sided *p*-value for T1m tests whether nAb titer associates with COVID-19 for any distance and for T2m tests whether the association with COVID-19 varies with viral distance.

**Fig. 5.**
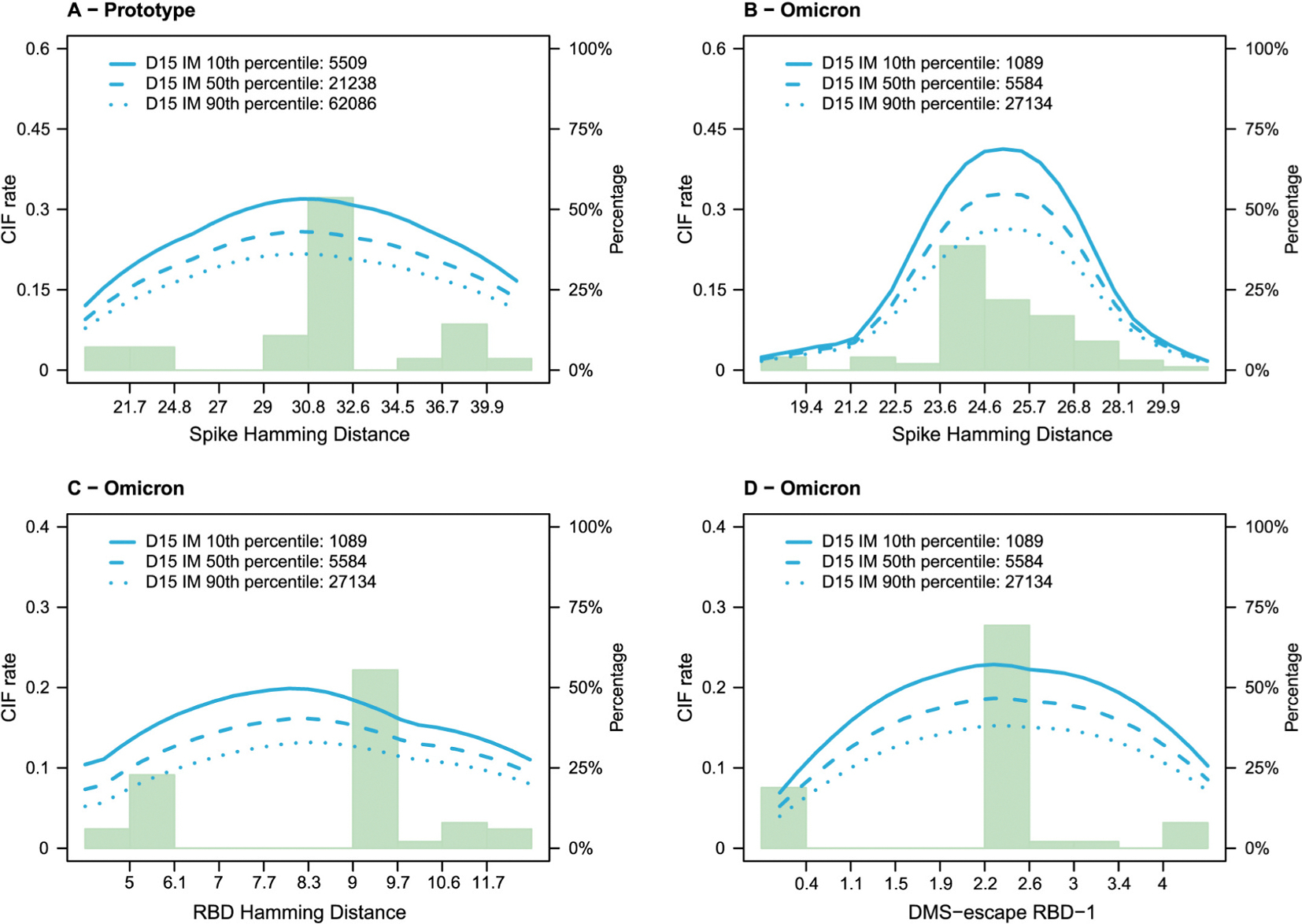
Distance-specific COVID-19 Cumulative Incidence Function (CIF) rates for D15 nAb titer markers set to the 10th, 50th, or 90th percentile values for the Prototype Vaccine Group and the Omicron Vaccine Group. For the Prototype Vaccine Group, nAb titer is against D614G. For the Omicron Vaccine Group, nAb titer is against BA.1 except for Arm 17 it is against BA.4/BA.5.

Figs. 4 and 5 show the analysis of D15 nAb titers as distance-specific CoRs for the Prototype and the Omicron Vaccine Groups, for the pooled Naïve + Non-naïve cohorts and all distances qualifying for analysis based on sufficient variability (Supplemental Figs. 3 and 4 restrict to the Naïve cohort). In Fig. 4, while the small 2-sided *p*-values from the test statistic T1m indicated evidence that titer inversely correlates with COVID-19, our central interest is in the 2-sided p-values for T2m that test whether the correlate of risk weakens against viruses with greater viral distance. The p-values for T2m ranged from 0.16 to 0.49, not rejecting the null hypothesis. However, the smallest p-value (0.16) was for the distance with greatest variability and hence greatest power in the analysis (RBD Hamming distance), and the estimate of the distance-specific hazard ratio showed the trend with the strongest correlate against the shortest-distance viruses (HR = 0.60, 95% CI 0.39–0.92) and the weakest correlate against the longest-distance viruses (HR = 0.77, 95% CI 0.59–1.02). Fig. 5 shows the correlates results in a different way, showing the distance-specific Cumulative Incidence Function (CIF) rate for the value of D15 nAb titer fixed at the 10th, 50th, or 90th percentile value. The estimated curves being ordered from top to bottom moving from 10th to 50th to 90th percentile reveals the overall correlate of risk result also revealed by T1m as noted above. For our salient question, when comparing the CIF rate curves at the 10th and 90th percentile titers, we are looking to see whether the 10th vs. 90th percentile curves have greater departure at small distances than at large distances. This was potentially the case for RBD Hamming distance (Fig. 5C) but not for Spike Hamming distance (Fig. 5A, B) or DMS-escape RBD-1 (Fig. 5D).

Restricting to Naïve participants, the results were similar to those for pooled Naïve and Non-naïve participants (Supplementary Figs. 3 and 4). For example, the p-value for the CoR varying by RBD Hamming distance was 0.18 in Naïve participants (Supplementary Fig. 3C) compared to 0.16 in Naïve and Non-naïve participants pooled (Fig. 4C).

## 5. Discussion

Zhang et al. [34] showed that the nAb ID50 titer measured two-weeks after a second booster dose with a Moderna or Pfizer-BioNTech mRNA vaccine correlated with a lower risk of COVID-19, but did not investigate how SARS-CoV-2 Spike amino acid sequences influenced the immune correlate. This sequel project considered five Spike or RBD amino acid sequence metrics quantifying dissimilarity of the SARS-CoV-2 sequences causing COVID-19 endpoints to the nearest vaccine-insert sequence or the XBB.1.5 reference sequence, to evaluate whether and how the previously documented nAb titer correlate of risk depended on the sequence metrics. With precedents from HIV-1 and dengue vaccines [43,51], our hypothesis was that the correlate of risk would weaken against SARS-CoV-2 strains with greater distances, and testing this hypothesis for several sequence metrics would provide an in vivo technique for discerning which metrics were immunologically significant. Our main result is lack of evidence that the correlate of risk weakened with viral distance, with *p*-values >0.10 for the 10 inferential analyses that were conducted, where this result is consistent with the previous result in COVAIL that BA.4/BA.5 titer was not a superior correlate of BA.4/BA.5 COVID-19 compared to D614G titer [34].

We propose that the negative result can be explained by limited genetic and antigenic dynamic range of the sequence distances. Indeed, for the Omicron Vaccine Group the first two deep mutational scanning escape RBD distances of case-causing viruses had median values 1.73 and 1.57, respectively, with ranges from 0 to 3.02 and 0 to 2.87. These values reflect the fold-difference in neutralization sensitivity of two viral isolates to sera from XBB.1.5-strain infected persons, where the previous immune correlates analysis of COVAIL one-dose mRNA Omicron-containing second-booster recipients estimated that a 10-fold change in nAb titer corresponded to a COVID-19 hazard ratio of 0.77 for Naïve and 0.52 for Non-naïve participants (Fig. 4 in [34]). In contrast, a mere 3-fold change in nAb titer corresponds to a COVID-19 hazard ratio of 0.88 for Naïve and 0.73 for Non-naïve participants. These modest effect sizes translate into relatively low power to detect modification of the nAb titer correlate of risk by viral sequence distance in the context of COVAIL. Future correlates analyses may need to pre-specify thresholds of sequence variability or focus on periods of strain turnover to optimize signal detection. Additional support for our proposed explanation stems from the finding that the smallest p-values (*p* = 0.16, 0.18) were for the sequence metric with greatest dynamic range – the RBD Hamming distance – with the point estimate hazard ratio per 10-fold change in D15 nAb titer changing from 0.60 to 0.77 against viruses with the shortest and longest distances to the Omicron-insert sequence, respectively. This suggests that a study with more participants or including more antigenically diverse viruses might demonstrate weakening of the correlate of risk with increasing distance. RBD Hamming distance has been previously shown to associate with vaccine efficacy, which diminishes against viruses more distant to the vaccine strain [36,52], although the trial-level analysis [52] was implemented before Omicron emerged when the range of RBD amino acid mismatches to the vaccine strain was only 0–3 compared to 0.4–18.6 mismatches in the present study. Moreover, 99 of the 101 (98.0%) COVID-19 endpoint cases in the Omicron Vaccine Group had an exact AA sequence match to XBB.1.5 in the 14 greatest-escape positions comprising the DMS-escape RBD-3 distance [44]. These frequencies highlight the limited antibody escape from XBB.1.5 among the circulating viral sequences that caused infections in COVAIL.

In addition to its widest dynamic range, the RBD Hamming distance had greater sensitivity to detect a sequence distance-dependent correlate of risk than the antibody escape score distances because the Hamming distances were calculated to the (nearest) vaccine-insert sequence as reference whereas the escape score distances used the XBB.1.5 sequence as reference, and the nAb titer utilized in the analysis was measured to the vaccine-insert sequence but not against XBB.1.5. The most statistically powerful version of the analysis uses a vaccine-insert reference sequence for the study endpoint sequence distances paired to measurement of the immune response against the vaccine-insert antigen, given that vaccines elicit their highest responses against homologous sequences, and this ideal analysis was conducted for the aforementioned HIV-1 and dengue analyses that detected signals. The ideal antibody escape score analysis would have run the deep mutational scanning experiments for each of the two Omicron vaccine-insert sequences BA.1 and BA.4/BA.5. However, since these experimental data were not available, due to the resource-intensity of deep mutational scanning experiments, the best available data were used instead, which were derived from the circulating Omicron variant XBB.1.5. The closer XBB.1.5 matches the Omicron vaccine-insert strains, the less noise in the analysis and the greater approximation of the implemented analysis to the ideal analysis. The previous antigenic cartography analysis showed that Stage 1 Moderna vaccinee sera at D15 had a geometric mean nAb titer against XBB.1.5 about 10-fold lower than against BA.1 or BA.4/BA.5 [23], indicating substantial noise in the implemented analysis.

We consider limitations of this study. First, as noted above the genetic and antigenic variability of circulating strains during the 13-month calendar follow-up period limited statistical power for detecting distance-specific correlates of risk. This limitation underscores the significance of studying the dynamic range of circulating strain characteristics over a given calendar period in a given population for informing various decisions such as the timing of updating booster strains. Second, as stressed above, the deep mutational scanning experimental data were available for the XBB.1.5 reference strain but not for each Omicron vaccine-insert strain (BA.1, BA.4/BA.5) – availability of these data would have enhanced the precision and power for detecting whether the nAb titer correlate of risk weakened with escape score distance. Third, there were insufficient data to study distance-specific correlates of risk separately by vaccine manufacturer and separately for Non-naïve participants, with the latter limitation significant given that the nAb titer correlate of risk was stronger in Non-naïve participants [34]. Fourth, ideally a study would evaluate immune correlates for severe/hospitalized COVID-19 for maximal clinical significance, which was not possible given the rarity of this outcome in a fully immunized study population. Fifth, 34% of the COVID-19 endpoint cases were missing viral sequences, necessitating statistical methods to account for this gap. Sixth, this study only evaluated nAb titer, and other immune responses could be playing a larger role in protecting against variant COVID-19 perhaps negating to see associations with titer. The literature is replete with results supporting that low neutralizing antibody titer marks vulnerability to COVID-19, and some literature also support that certain high-quality and quantity T cell responses may be able to compensate for this vulnerability (e.g., the “swiss cheese model” [53]). Accordingly, had sufficient T cell data been available from COVAIL participants, it would be interesting to investigate whether the nAb titer distance-specific correlate of risk had stronger attenuation with distance for individuals with poor T cell response. Lastly, given that neutralizing epitopes of SARS-CoV-2 are generally conformational rather than linear, and the distances considered were not designed to target conformational epitopes, future research could pursue more structurally/conformationally relevant viral distances.

Strengths of this research project include a large number of COVID-19 endpoints and use of a validated pseudovirus neutralization assay conducted against multiple strains including those in the vaccines tested as well as those in circulation during the study. While 34% of the COVID-19 endpoint cases were missing viral sequences as stated above, an additional strength of the present study is that we designed statistical methods to account for this fact, with predictive mean matching/hotdeck imputation performed to limit bias and improve efficiency.

In conclusion, these findings underscore the value of integrating viral sequence data into immune correlate analyses, while also highlighting the challenge of doing so when there is limited antigenic diversity among circulating strains during the trial period. Specifically, the genetic and antigenic heterogeneity in the United States from April 2022 to May 2023 during the correlates analysis follow-up period of COVAIL appeared to be too limited to alter the nAb titer correlate of risk, reflecting only an approximately 3-fold range of differential nAb titer sensitivity to sera. Additionally, the trend of the neutralizing antibody titer correlate of risk being stronger against strains better matched in their RBD amino acid sequence to their vaccine-insert sequences is likely a real result predicting that if there had been a substantially greater dynamic range of circulating sequences than this study would have detected it. These results inform deliberations about the extent of genetic and antigenic-specificity of SARS-CoV-2 viral evolution that must occur before updating the booster-strain is predicted to substantially influence the level of booster-protection.

## Supplementary Material

MMC1

## Figures and Tables

**Table 1:** COVAIL trial participants included in the correlates analyses.

	Naïve Cohort				Non-naïve Cohort				Naïve + Non-naïve Pooled			
	COVID-19 Cases		Non-cases		COVID-19 Cases		Non-cases		COVID-19 Cases		Non-cases	
						
Vaccine Group*	N (w/ seqs)	GM (95% CI)	N	GM (95% CI)	N (w/ seqs)	GM (95% CI)	N	GM (95% CI)	N (w/ seqs)	GM (95% CI)	N	GM (95% CI)

Prototype (Arms 1, 7)	36 (25)	15,572 (12,172, 19922)	69	16,522 (13,335, 20472)	7 (3)	32,670 (21,154, 50456)	31	47,539 (34,775, 64988)	43 (28)	17,568 (13,983, 22072)	100	22,927 (18,786, 27982)
Moderna Prototype (Arm 1)	23 (17)	15,801 (11,397, 21907)	52	17,523 (13,486, 22769)	4 (2)	35,865 (NA, NA)	17	46,930 (29,310, 75142)	27 (19)	17,841 (13,093, 24312)	69	22,337 (17,476, 28550)
Pfizer-BioNTech Prototype (Arm 7)	13 (8)	15,174 (9903, 23251)	17	13,803 (9536, 19978)	3 (1)	28,849 (NA, NA)	14	48,289 (30,377, 76764)	16 (9)	17,117 (11,841, 24742)	31	24,299 (16,974, 34784)
Omicron (Arms 2, 4–6, 8–9, 12, 16, 17)**	130 (92)	3227 (2678, 3889)	329	3902 (3362, 4529)	22 (9)	5592 (3376, 9262)	263	10,540 (9136, 12159)	152,101)	3495 (2931, 4167)	592	6068 (5428, 6783)
Moderna Beta+Omicron BA.1 (Arm 2)	32 (22)	3396 (2288, 5041)	54	4365 (3038, 6273)	5 (3)	14,121 (1973, 101058)	18	16,143 (6725, 38752)	37 (25)	4117 (2716, 6241)	72	6054 (4211, 8702)
Moderna Delta+Omicron BA.1 (Arm 4)	25 (20)	3621 (2311, 5673)	58	3891 (2479, 6108)	3 (2)	8229 (201, 336805)	12	12,348 (4942, 30851)	28 (22)	3954 (2548, 6136)	70	4743 (3149, 7145)
Moderna Omicron BA.1 (Arm 5)	24 (17)	3409 (2166, 5365)	49	5260 (3553, 7786)	1 (0)	7413 (NA, NA)	22	14,886 (10,992, 20159)	25 (17)	3517 (2268, 5453)	71	7260 (5355, 9844)
Moderna Omicron BA.1 + Prototype (Arm 6)	23 (15)	2555 (1556, 4195)	49	4989 (3659, 6803)	3 (2)	3992 (1264, 12608)	17	25,271 (16,584, 38507)	26 (17)	2690 (1730, 4184)	66	7577 (5587, 10277)
Pfz Beta+Omicron BA.1 (Arm 8)	7 (4)	4150 (1586, 10859)	26	2264 (1561, 3283)	0 (0)	NA (NA, NA)	17	8466 (3468, 20668)	7 (4)	4150 (1586, 10859)	4	3813 (2447, 5942)
Pfizer-BioNTech Omicron BA.1 (Arm 9)	5 (3)	2194 (511, 9421)	29	3703 (2254, 6085)	3 (0)	1946 (1473, 2570)	15	12,652 (6717, 23831)	8 (3)	2097 (996, 4419)	44	5630 (3709, 8544)
Pfizer-BioNTech Omicron BA.1 + Prototype (Arm 12)	5 (4)	3273 (950, 11271)	29	4063 (2441, 6761)	1 (1)	3655 (NA, NA)	17	11,277 (5694, 22335)	6 (5)	3334 (1308, 8500)	46	5925 (3896, 9009)
Pfizer-BioNTech Omicron BA.1 + Prototype (Arm 16)	5 (4)	3692 (952, 14317)	15	2610 (1260, 5405)	3 (0)	5515 (2460, 12366)	75	8331 (6594, 10524)	8 (4)	4292 (2077, 8870)	90	6865 (5397, 8733)
Pfizer-BioNTech Omicron BA.4/BA.5 + Prototype (Arm 17)	4 (3)	2501 (401, 15611)	20	2142 (1058, 4337)	3 (1)	3475 (198, 60851)	70	8560 (6757, 10844)	7 (4)	2880 (1075, 7716)	90	6291 (4834, 8189)

* For Prototype Vaccine Group arms, nAb titer is against D614G. For Omicron Vaccine Group arms, nAb titer is against BA.1 for all arms except Arm 17 for which it is against BA.4/BA.5. Follow-up for correlates (7 days post D15 through 188 days post D15) occurred from April 21, 2022 to May 25, 2023. Mod, Moderna mRNA. Pfz, Pfizer-BioNTech mRNA.

** Omicron vaccine-insert sequence is Omicron BA.1 for Arms 2, 4–6, 8–9, 12, 16; and BA.4/BA.5 for Arm 17.

**Table 2:** Viral sequence-distance specific correlates of risk analyses conducted*

Vaccine Group	Immunity Group	D15 nAb ID50 Marker	Viral Sequence Distance

Prototype Vaccine Group	Naïve+Non-naïve	D614G	spike weighted Hamming Distance
	Naïve	D614G	spike weighted Hamming Distance
Omicron Vaccine Group	Naïve+Non-naïve	BA.1 for arms 2, 4–6, 8–9, 12, 16; BA.4/BA.5 for arm 17	spike weighted Hamming Distance
		BA.1 for arms 2, 4–6, 8–9, 12, 16; BA.4/BA.5 for arm 17	RBD weighted Hamming Distance
		BA.1 for arms 2, 4–6, 8–9, 12, 16; BA.4/BA.5 for arm 17	DMS-escape RBD-1, RBD-2, RBD-3
	Naïve	BA.1 for arms 2, 4–6, 8–9, 12, 16; BA.4/BA.5 for arm 17	spike weighted Hamming Distance
		BA.1 for arms 2, 4–6, 8–9, 12, 16; BA.4/BA.5 for arm 17	RBD weighted Hamming Distance
		BA.1 for arms 2, 4–6, 8–9, 12, 16; BA.4/BA.5 for arm 17	DMS-escape RBD-1, RBD-2, RBD-3

* DMS-escape RBD-1 and RBD-2 are quantitative distances including all RBD amino acid (AA) positions and 48 relatively high-escape RBD AA positions, respectively. These 48 positions are: 335, 337, 339, 344, 346, 347, 348, 352, 356, 357, 359, 363, 365, 367, 368, 371, 375, 376, 385, 393, 408, 420, 421, 428, 439, 440, 444, 445, 447, 448, 449, 450, 451, 452, 453, 455, 456, 462, 472, 473, 475, 478, 483, 485, 487, 490, 518, 520.

**Table 3:** Baseline demographics of COVAIL trial participants included in the correlates analyses.

		N (Percent) or Geometric Mean (range)					
	
Vaccine Group*	Variable	Naïve Cohort	Naïve Cases	Non-naïve Cohort	Non-naïve Cases	Naïve + Non-naïve Pooled	Naïve + Non-naïve Cases

Prototype (Arms 1, 7)	Age	65 (61.9%) 18–64 40 (38.1%) ≥65	26 (72.2%) 18–64 10 (27.8%) ≥65	30 (78.9%) 18–64 8 (21.1%) ≥65	7 (100%) 18–64 0 (0%) ≥65	95 (66.4%) 18–64 48 (33.6%) ≥65	33 (76.7%) 18–64 10 (23.3%) ≥65
	Sex	51 (48.6%) female 54 (51.4%) male	17 (47.2%) fem. 19 (52.8%) male	21 (55.3%) fem. 17 (44.7%) male	5 (71.4%) fem. 2 (28.6%) male	72 (50.3%) fem. 71 (49.7%) male	22 (51.2%) fem. 21 (48.8%) male
	Vaccination history	56 (53.3%) P, P 39 (37.1%) M, M 0 (0%) J, J 5 (4.8%) P, M 3 (2.9%) M, P 0 (0%) J, P 2 (1.9%) J, M	23 (63.9%) P, P 11 (30.6%) M, M 0 (0%) J, J 0 (0%) P, M 1 (2.8%) M, P 0 (0%) J, P 1 (2.8%) J, M	17 (44.7%) P, P 15 (39.5%) M, M 0 (0%) J, J 1 (2.6%) P, M 3 (7.9%) M, P 1 (2.6%) J, P 1 (2.6%) J, M	6 (85.7%) P, P 0 (0%) M, M 0 (0%) J, J 0 (0%) P, M 0 (0%) M, P 1 (14.3%) J, P 0 (0%) J, M	73 (51%) P, P 54 (37.8%) M, M 0 (0%) J, J 6 (4.2%) P, M 6 (4.2%) M, P 1 (0.7%) J, P 3 (2.1%) J, M	29 (67.4%) P, P 11 (25.6%) M, M 0 (0%) J, J 0 (0%) P, M 1 (2.3%) M, P 1 (2.3%) J, P 1 (2.3%) J, M
	nAb titer D614G	16,190 (2205, 68899)	15,572 (2633, 59225)	44,365 (12,073, 373292)	32,670 (15,715, 64438)	21,163 (2205, 373292)	17,568 (2633, 64438)
	nAb titer BA.4/5	787 (26, 14228)	776 (91, 3601)	2904 (373, 64912)	1870 (631, 3743)	1113 (26, 64912)	896 (91, 3743)
Omicron (Arms 2, 4–6, 8–9, 12, 16, 17)	Age	302 (65.8%) 18–64 157 (34.2%) ≥65	102 (78.5%) 18–64 28 (21.5%) ≥65	257 (90.2%) 18–64 28 (9.8%) ≥65	21 (95.5%) 18–64 1 (4.5%) ≥65	559 (75.1%) 18–64 185 (24.9%) ≥65	123 (80.9%) 18–64 29 (19.1%) ≥65
	Sex	247 (53.8%) female 212 (46.2%) male	75 (57.7%) fem. 55 (42.3%) male	160 (56.1%) fem. 125 (43.9%) male	14 (63.6%) fem. 8 (36.4%) male	407 (54.7%) fem. 337 (45.3%) male	89 (58.6%) fem. 63 (41.4%) male
	Vaccination history*	228 (49.7%) P, P 150 (32.7%) M, M 1 (0.2%) J, J 37 (8.1%) P, M 24 (5.2%) M, P 5 (1.1%) J, P 14 (3.1%) J, M	66 (50.8%) P, P 39 (30%) M, M 0 (0%) J, J 14 (10.8%) P, M 6 (4.6%) M, P 0 (0%) J, P 5 (3.8%) J, M	159 (55.8%) P, P 80 (28.1%) M, M 4 (1.4%) J, J 17 (6%) P, M 14 (4.9%) M, P 5 (1.8%) J, P 6 (2.1%) J, M	10 (45.5%) P, P 5 (22.7%) M, M 1 (4.5%) J, J 4 (18.2%) P, M 1 (4.5%) M, P 1 (4.5%) J, P 0 (0%) J, M	387 (52%) P, P 230 (30.9%) M, M 5 (0.7%) J, J 54 (7.3%) P, M 38 (5.1%) M, P 10 (1.3%) J, P 20 (2.7%) J, M	76 (50%) P, P 44 (28.9%) M, M 1 (0.7%) J, J 18 (11.8%) P, M 7 (4.6%) M, P 1 (0.7%) J, P 5 (3.3%) J, M
	nAb titer D614G	18,504 (20, 389237)	16,137 (1917, 162927)	34,946 (20, 463855)	22,529 (3013, 166422)	23,607 (20, 463855)	16,935 (1917, 166422)
	nAb titer BA.4/5	1418 (20, 54695)	1171 (20, 54695)	5180 (26, 229463)	2674 (200, 26138)	2329 (20, 229463)	1320 (20, 54695)

* Measures the primary series vaccine type (2 doses of Pfizer-BioNTech mRNA, 2 doses of Moderna mRNA, 1 dose of Janssen Ad26.COV2·S) and the first boost type (Pfizer-BioNTech, Moderna, or Janssen). P, P = Pfizer-BioNTech 2 dose primary series and Pfizer-BioNTech first boost; M, M = Moderna 2 dose primary series and Moderna first boost; J, J = Janssen 1 dose primary series and Janssen first boost, and so on.

**Table 4:** For the Omicron Vaccine Group: Motifs of COVID-19 endpoint sequences at the 14 RBD amino acid positions with greatest neutralization escape mutations 352, 357, 371, 375, 420, 421, 440, 447, 450, 455, 456, 473, 475, 485 comprising the DMS-escape RBD-3 distances (Fig. 4a in ref. [44])*

RBD-3 AA Motif^1^	XBB.1.5 antibody escape score	Frequency for COVID-19 endpoints	COVAIL Vaccine-Insert Strains

ARFFDYKGNLFYAG	0 (XBB.1.5 sequence)	99 (98.0%)	BA.4/BA.5
ARFFDYKGDLFYAG	2.84231	1 (1.0%)	None
ARFFDYKGNLLYAG	4.21352	1 (1.0%)	None

* The table also indicates the motifs for the three vaccine-insert sequences in the Omicron Vaccine Group.

1 Amino acids indicated in boldface are those that do not match the XBB.1.5 wild type residue for which the escape score is zero.

## Data Availability

The trial dataset will be available to appropriate academic parties on request from the corresponding author, in accordance with the data sharing policies of the Division of Microbiology and Infectious Diseases of NIAID with input from the investigator group subject to submission of a suitable study protocol and analysis plan. Requests should be directed to Dr. Paul C. Roberts (paul.roberts@nih.gov). The SARS-CoV-2 sequence data analyzed in this work are available in the BioProject database under accession number PRJNA1301408.

## Code availability

All analyses were done reproducibly on the basis of publicly available R scripts hosted on the GitHub collaborative programming platform (https://github.com/fei-heng/COVAIL_reporting).

All authors attest they meet the ICMJE criteria for authorship.

## Appendix A. Supplementary data

Supplementary data to this article can be found online at https://doi.org/10.1016/j.vaccine.2026.128348.
