# Supplementary material for: The neutralizing antibody titer correlate of COVID-19 risk in the COVID-19 variant immunologic landscape (COVAIL) trial was not modified by SARS-CoV-2 amino acid sequence distances
Source: Vaccine. Author manuscript; Available in PMC 2026 Mar 23. (PMC13008134; doi:10.1016/j.vaccine.2026.128348)
Supplement: MMC1 [file NIHMS2150017-supplement-MMC1.docx]

**Supplementary Material for “The neutralizing antibody titer correlate of COVID-19 risk in the COVAIL trial was not modified by SARS-CoV-2 amino acid sequence distance”**

Contents

[COVAIL Manuscript Study Group 2](#_Toc212793752)

[COVAIL Manuscript Study Team Members 5](#_Toc212793753)

[Supplementary Methods 10](#_Toc212793754)

[Supplementary Figure 1: Correlation of the Spike and RBD physico-chemical weighted Hamming distances for the Prototype Vaccine Group. 13](#_Toc212793755)

[Supplementary Figure 2: Restricted to the Naïve cohort. For the viral sequence distances studied in immune correlates analyses, viral distances vs. Day 15 nAb ID50 titer for COVID-19 endpoint cases for the Prototype Vaccine Group and the Omicron Vaccine Group. 14](#_Toc212793756)

[Supplementary Figure 3: Restricted to the Naïve cohort. Hazard ratios of viral distance-specific COVID-19 for Day 15 nAb ID50 titer markers for the Prototype Vaccine Group and the Omicron Vaccine Group. 15](#_Toc212793757)

[Supplementary Figure 4: Restricted to the Naïve cohort. Distance-specific COVID-19 Cumulative Incidence Function rates for Day 15 nAb ID50 titer markers set to the 10th, 50th, or 90th percentile values for the Prototype Vaccine Group and the Omicron Vaccine Group. 16](#_Toc212793758)

# COVAIL Manuscript Study Group

**George Washington University, Washington D.C.**

David J. Diemert, MD; Elissa Malkin, DO; Jeffrey M. Bethony, PhD; Aimee Desrosiers, PA-C; Marc Siegel, MD

**University of Rochester VTEU, Rochester, NY**

Angela R. Branche, MD; Ann R. Falsey, MD; Edward Walsh, MD; Patrick Kingsley, BS; Michael Peasley, BS

**Emory University Hope Clinic, Decatur, GA**

Nadine G. Rouphael, MD; Cecilia Losada, MD; Daniel S. Graciaa, MD; Hady Samaha, MD; Paulina A Rebolledo, MD; Zanthia Wiley, MD.

**Brigham and Women’s Hospital, Harvard Medical School, Boston, MA**

Lindsey R. Baden, MD; Amy C. Sherman, MD; Stephen R. Walsh, MD; Alexandra Tong, BS; Rebecca Rooks, BS

**Saint Louis University, St. Louis, MO**

Sharon E. Frey, MD; Getahun Abate, MD, PhD; Zacharoula Oikonomopoulou, MD; Daniel F. Hoft, MD, PhD; Irene Graham, MD

**Departments of Molecular Virology and Microbiology and Medicine, Baylor College of Medicine, Houston, TX**

Jennifer A. Whitaker, MD; Hana M. El Sahly, MD; Wendy A. Keitel, MD; C. Mary Healy, MD; Robert L. Atmar, MD

**Department of Medicine, Division of Infectious Diseases and Global Public Health, University of California San Diego, La Jolla, CA**

Susan J. Little, MD; Thomas C.S. Martin, MD; Nicole Carter, MPH; Steven Hendrickx, RN

**Center for Childhood Infections and Vaccines (CCIV) of Children’s Healthcare of Atlanta and Emory University Department of Pediatrics, Atlanta, GA**

Evan J. Anderson, MD; Christina A. Rostad, MD; Satoshi Kamidani, MD; Etza Peters, RN

**Duke Human Vaccine Institute, Duke University School of Medicine, Durham, NC**

Emmanuel B. Walter, MD, MPH; Michael J. Smith, MD, MSCE; M. Anthony Moody, MD; Kenneth E. Schmader, MD

**University of Illinois at Chicago-Project WISH, Chicago, IL**

Richard M. Novak, MD; Benjamin G. Ladner, MD; Andrea Wendrow, RPh; Jessica Herrick, MD

**University of Texas Medical Branch, League City, TX**

Richard Rupp, MD; Laura Porterfield, MD

**Kaiser Permanente Washington Health Research Institute, Seattle, WA**

Lisa A. Jackson, MD, MPH; Maya Dunstan, MS, RN; Rebecca Lau, PharmD; Barbara Carste, MPH

**Department of Medicine, University of Washington, Seattle, WA**

Tara M. Babu, MD, MSCI; Anna Wald, MD, MPH; Taylor Krause, BA; Kirsten Hauge, MPH

**NYU VTEU Manhattan Research Clinic at NYU Grossman School of Medicine, New York, NY**

Angelica C. Kottkamp, MD; Mark J. Mulligan, MD; Tamia Davis, NP; Celia Engelson, NP; Vijaya Soma, MD

**Zuckerberg San Francisco General, University of California San Francisco, San Francisco, CA**

Anne F. Luetkemeyer, MD; Chloe Harris, BA; Azquena Munoz Lopez, BS

**Morehouse School of Medicine, Atlanta, GA**

Lilly C. Immergluck, MD; Erica Johnson, PhD; Austin Chan, MD

**Washington University School of Medicine, St. Louis, MO**

Rachel M. Presti, MD, PhD; Jane A. O’Halloran, MD, PhD; Ryley M. Thompson

**NYU VTEU Long Island Research Clinic at NYU Long Island School of Medicine, Mineola, NY**

Martín Bäcker, MD; Andrew B. Fleming, MD; Asif Noor, MD

**University of Iowa College of Medicine, Iowa City, IA**

Patricia L. Winokur, MD; Jeffery Meier, MD; Jack Stapleton, MD

**Howard University College of Medicine, Howard University Hospital, Washington D.C.**

Siham M. Mahgoub, MD; Celia Maxwell, MD; Sarah Shami, PharmD

**University of Alabama at Birmingham, Birmingham, AL**

Paul A. Goepfert, MD

**Tulane University School of Medicine, New Orleans, LA**

Dahlene N. Fusco, MD; Arnaud C. Drouin, MD; Florice K. Numbi, MD

**University of Maryland, Baltimore, MD**

Kirsten E. Lyke, MD

**IDCRC Principal Investigators**

David S. Stephens, MD; Kathleen M. Neuzil, MD

**IDCRC Leadership Operations Center**

Monica M. Farley, MD; Jeanne Marrazzo, MD; Sidnee Paschal Young

**IDCRC Clinical Operations Unit**

Jeffery Lennox, MD; Robert L. Atmar, MD; Linda McNeil FHI360

**IDCRC Statistical and Data Science Unit**

Elizabeth Brown, PhD

**IDCRC Laboratory Operations Unit – Fred Hutchinson Cancer Center, Seattle, WA**

Christine M. Posavad, PhD; Megan A. Meagher, BS; Julie McElrath, MD; Mike Gale, PhD

**FHI360, Durham, NC**

Kuleni Abebe, MSc

**The Emmes Company, LLC, Rockville, MD**

Mat Makowski, PhD; Heather Hill, MS; Jim Albert, MS; Holly Baughman; Lisa McQuarrie, MS; Kalyani Telu, MS; Jinjian Mu, PhD; Lisa J. McQuarrie^,^ MSc

**Clinical Monitoring Research Program Directorate, Frederick National Laboratory for Cancer Research, Frederick, MD**

Teri C. Lewis, BS; Lisa A. Giebeig, MS; Theresa M. Engel, MFS; Caleb J. Griffith, MPH; Wendi L. McDonald, BSN; Alissa E. Burkey, MS; Lisa B. Hoopengardner, MS; Jessica E. Linton, MS; Nikki L. Gettinger, MPH

**Department of Surgery and Duke Human Vaccine Institute, Duke University School of Medicine, Durham, NC**

David C. Montefiori, PhD; Amanda Eaton, MBA

**Smith’s Laboratory, Cambridge, UK**

Derek J. Smith, PhD; Antonia Netzl; Samuel H. Wilks, PhD; Sina Türeli, PhD

**Division of Microbiology and Infectious Diseases, National Institute of Allergy and Infectious Diseases, National Institutes of Health, Bethesda, MD.**

Mamodikoe Makhene, MD; Mohamed Elsafy, MD; Rhonda Pikaart-Tautges, BS; Janice Arega, MS; Binh Hoang, RPh; Dan Curtin; Hyung Koo, BSN; Elisa Sindall, BSN; Sonja Crandon, BSN; Marciela M. DeGrace, PhD; Diane J. Post, PhD; Seema U. Nayak, MD; Paul C. Roberts, PhD; John H. Beigel, MD

# COVAIL Manuscript Study Team Members

**Emory University Hope Clinic, Decatur, GA**

Nadine G. Rouphael, MD; Cecilia Losada, MD; Daniel S. Graciaa, MD; Hady Samaha, MD; Cassie Grimsley Ackerley, MD; Kristen E. Unterberger, PA; Amy Anderson, BSN; Mary Atha, ACNP; Kareem Bechnak, BSN; Sarah Bechnak, BSN; Mary Bower, BSN; Laura Clegg, RN; Matthew Collins, MD, PhD; Francine Dyer, RN; Srilatha Edupuganti, MD; Rebecca Fineman, BS; Tigisty Girmay, MSN; Rebecca Gonzalez, PharmD; Natalie Gray, BS; Evan Gutter, MPH; Lisa Harewood; Chris Huerta, MSc; Brandi Johnson, BS; Lauren Johnson, MPH; Colleen Kelley, MD; Alexandra Koumanelis, BA; Deborah Laryea, BSN; Hollie Macenczak, BSN; Nour Makkaoui, MD; Michele McCullough, MPH; Tuong-Vy Ngo, PharmD; Eileen Osinski, BS; Julia Paine, BS; Bernadine Panganiban, BS; Rose Pope, RN; Paulina Rebolledo, MD; Susan Rogers, RPh; Erin Scherer, PhD; Veronica Smith, NP-C; Andre Stringer, BS; Jessica Traenkner, PA; Dongli Wang, BS; Alahna Watson, BA; Stacey Wheeler, RN; Jean Winter; Jianguo Xu, PhD

**Brigham and Women’s Hospital, Harvard Medical School, Boston, MA**

Lindsey R. Baden, MD; Amy C. Sherman, MD; Stephen R. Walsh, MD; Alexandra Tong, BS; Rebecca Rooks, BS; Jane A. Kleinjan, NP; Jon A. Gothing, NP; Andres A. Avila Paz, BA; Muneerah M. Aleissa, PharmD, MPH; Bethany Evans, BA; August Heithoff, BS; Natalie E. Izaguirre, MS; Hannah Jin, MPH; Urwah Kanwal, BS; Austin Kim, BS; Julia E. Klopfer, BS; Christina Montesano, BS; John Almeida, BA; Emily S. Koleske, BS; Hannah Levine, BS; Nicholas P. Morreale, BS; Omolola Ometoruwa, BS; Jun Bai Park Chang, BS; Anna F. Piermattei, BA; Djenane M. Pierre, BS; Megan Powell, BA; Kevin Zinchuk, PharmD; Stephanie Pickford, PharmD; Charles M. Kelly III, PharmD; Xiaofang Li, PhD; John Kupelian, BS ; Kimberly Dufresne, BS; Xiaoguang Fan, MD, PhD; Xi Zhang, PhD; Esther Arbona-Haddad, MD; Jose Humberto Licona, MD

**Center for Childhood Infections and Vaccines (CCIV) of Children’s Healthcare of Atlanta and Emory University Department of Pediatrics, Atlanta, GA**

Evan J. Anderson, MD; Christina A. Rostad, MD; Satoshi Kamidani, MD; Etza Peters, RN; Larry Anderson, MD; Julia Bartol; Leisa Bower, RN; Natsuko Campbell, RN; Lisa Harewood; Hui-Mien Hsiao; Laila Hussaini, MPH; Inara Jooma; Gidget Kettle, RN; Marcia Lewis, RN; Wensheng Li; Cindy Lubbers, RN; Lisa Macoy, RN; Molly Morrison, Heather Nurse, RN; Anna Siaw-Anim; Kathleen Stephens, RN; Madeline Taylor; Ashley Tippett, MPH; Lauren Nolan, PA

**Zuckerberg San Francisco General, University of California San Francisco, San Francisco, CA**

Anne F. Luetkemeyer, MD; Chloe Harris, BA; Azquena Munoz Lopez, BS; Daniel Berrner; Dennis Dentoni-Lasofsky, MSN; John Dwyer, RN; Suzanne Hendler, BSN; Elvira Gomez, MPH; WeyLing Phuah, PharmD; Jaime Velasco, BA; Veronica Viar, MS

**George Washington University, Washington D.C.**

David J. Diemert, MD; Elissa Malkin, DO; Jeffrey M. Bethony, PhD; Aimee Desrosiers, PA-C; Marc Siegel, MD; Nikita Schroll-McLaughlin, MS; Jonathan Manning, BA; Jane Ryu, MS; Hanna-Grace Rabanes, MPH; Khadija Khan, MPH; Laura Vasquez, MPH; Caroline Thoreson, PA-C; Larissa Scholte, PhD; Rafaela Thur, DVM; Peyton St. John, BS; Dorinne Mettle-Amuah, PharmD

**University of Iowa College of Medicine, Iowa City, IA**

Patricia L. Winokur, MD; Jeffery Meier, MD; Jack Stapleton, MD; Laura Stulken, PA; Theresa Hegmann, PA; Deb Pfab, RN; Elizabeth Morgan, RN; Susan Herman, RN; Angel Peguero, CMA; Michelle Rodenburg; Alfred J. Carr; Delilah Johnson

**Washington University School of Medicine, St. Louis, MO**

Rachel M. Presti, MD, PhD; Jane A. O’Halloran, MD, PhD; Michael Klebert, RN, PhD; Ryley M. Thompson; Alem Haile; Kim Gray, NP; Chapelle Ayres; Delaney Carani, RN; Michael Royal; John Tran; Laura Blair; Anita Afghanzada; Natalie Schodl

**NYU VTEU Manhattan Research Clinic at NYU Grossman School of Medicine, New York, NY**

Angelica C. Kottkamp, MD; Tamia Davis, NP; Celia Engelson, NP; Vijaya Soma, MD; Abdulwahab Abdulai; Ashanay Allen; Natella Aronova, NP; Philip Aziz, PharmD; Emily Beato; Samuel Bliss, PharmD; Jacqueline Callahan, RN; Ellie Carmody, MD; Amanda Dontino, BS; Aimee Edwin, RN; Shelby Goins; Sarah Haiken; Ramin Herati, MD; Abdonnie Holder; Janice Hong; Trishala Karmacharya; Manpreet Kaur, PharmD; Hye-Youn Kim; Alexander McMeeking, MD; Mark Mulligan, MD; Wai Ng; Edward Nirenberg; Irma Noriega, NP; Samuel Nweke; Lalitha Parameswaran, MD; Levonne Phillip, MPH; Stephanie Rettig, MPH; Marie Samanovic-Golden, PhD; Madalyn Saporito; Pamela Suman; Meron Tasissa; Michael Tuen; Julia Wagner, MPH; James Wilson; Doris Wong, PharmD; Grace Yip, BS; Samantha Yip, RN; Heekoung Youn, RN; Lisa Zhao

**University of Rochester VTEU, Rochester, NY**

Angela R. Branche, MD; Ann R. Falsey, MD; Edward E. Walsh, MD; Patrick Kingsley, BS; Arthur Zemanek, BSN, MS; Katherine Elena, BSN; Spencer Obrecht, BSN; Ian Shannon, BSN; Amy Kaychalo, BS, MS; Erin Nowicki; Sharon Moorehead; Kari Steinmetz, BA; Doreen Francis, RN; Tanya Smith, BS; William Hamilton, BS; Jeanne Holden-Wiltse, MPH, MBA; Christopher Lane, MS; Michael Peasley, BS; Samuel Diehl, BS; Kyle Richards, PharmD; Stephen Bean, PharmD; Nicole Dornbush, PharmD; Carol Cole, PharmD

**Saint Louis University, St. Louis, MO**

Sharon E. Frey, MD; Getahun Abate, MD, PhD; Zacharoula Oikonomopoulou, MD; Daniel F. Hoft, PhD, MD; Irene Graham, MD; Azra Blazevic, DVM, MPH; Tamara Blevins, MS; Kathleen Chirco, BSN; Sabrina M. DiPiazza, BSN, MA; Stanley Doublin; Heather Hoertel Douds, MSNS, BSN; Carol G. Duane, PhD, RN; Eric Eggemeyer, BA; Linda M. Eggemeyer-Sharpe, BSN; Lauren Nicole Foreman, BSN; Sarah Louise George, MD; Geoffrey J. Gorse, MD; Michelle Harris, PharmD; Helay Hassas, PharmD; Rong Hou, MD; Ryan Clark Kerr, BSN; Kate Elizabeth Liefer, BSN; Melissa J. Loyet, RN; Lainey Mejia-Jauregui, BS; Keith Meyer, BS; Tracy Renee Montauk, BSN; Karla J. Mosby, RN; Amanda Nethington, BS; Huan Ning, MD; Nicole Purcell; Joan M. Siegner, BSN, MA; Janice M. Tennant, BSN, MPH; Mei Xia, PhD; Kiana Wilder, BA; Yinyi Yu, BS; Cassandra Nicole Zehenny, BSN

**University of Texas Medical Branch, League City, TX**

Richard Rupp, MD; Laura Porterfield, MD; Amber Stanford, PA-C; Robert Cox, RN; Kristin Pollock, RN; Diane Barrett, MS; Gerrianne Casey, RN; Amy McMahan, LVN; Cori Burkett, PA-C; Essie Cox

**NYU VTEU Long Island Research Clinic at NYU Long Island School of Medicine, Mineola, NY**

Martín Bäcker, MD; Sarah J. Pastolero, RN; Kimberly Byrnes, RN; Andrew B. Fleming, MD; Asif Noor, MD; Sigridh A. Muñoz-Gómez, MD; Steven E. Carsons, MD; Sajumon K. Joseph, FNP; Sophie Danziger; Monica Benitez; Maung Aung; Louis Ragolia, PhD; Alicia Vasile, RPh; April Correll, RPh; Christopher Hall; Thomas Palaia; Miloni H Thakker, MD; Lavern Harvey; Lisa Zhao; Diana Badillo, MD.

**University of Illinois at Chicago-Project WISH, Chicago, IL**

Richard M. Novak, MD; Benjamin G. Ladner, MD; Andrea Wendrow, RPh; Jesica Herrick, MD; Alfredo J. Mena Lora, MD; Scott A. Borgetti, MD; Diana L Bahena, APRN; Regina Harden, BA; Renyce Powell;  David C. M. Chan, PharmD; Rebeca F. Gasari, PharmD; Michael Pacini, PharmD; Margarita M. Villarreal, CPhT; Rodrigo Reyes, ADN; Samuel M. Rene, MPH; Shannon M Whitted, BSN; Habiba Sultana, MBBS; Nanu Kunwar, BS; Tasmin Sultana, MBBS; Md R. Amin, PhD;  Mahmood Ghassemi, PhD,  Liam Morrissy, BS; Nia O'Neal, BS; Chasity Serrano, BS; Charlie Peterson, BA

**Duke Human Vaccine Institute, Duke University School of Medicine, Durham, NC**

Emmanuel B. Walter MD, MPH; Michael J. Smith MD, MSCE; M. Anthony Moody, MD; Kenneth E. Schmader, MD; Susan Doyle; Lynn S Harrington BSN; Lori Hendrickson BSN; Amy O’Berry MSN; Sherry Huber BSN; Janet Wootton RN, RSCN; Kelly Clark BA; Lani Banez; Stephanie Smith BA; Byron Hauser BS; Ally Odom BA; Emily Randolph BA; Krystina Yoder BA; Kathlene Chmielewski; Luis Ballon BA; Aubree Latorre; Breana Montgomery; Antony Tritz MS; Thad Gurley, MS; Margaret Pendzich

**Kaiser Permanente Washington Health Research Institute, Seattle, WA**

Lisa A. Jackson, MD, MPH; Maya Dunstan, MS, RN; Rebecca Lau, PharmD; Barbara Carste, MPH; Wesley A. Andersen, RPh, MHA, MA; Lee Barr, RN; Cassandra Bryant, BS; Joe Choe, BS; Lynn Gross, PA-C; Erika Kiniry, MPH; Bonnie Y Lam, PharmD; De Vona Lang; Stella Lee, BA; Paula J Lins, PA-C, MPH; Amy Mohelnitzky, PA-C; Marilyn Nguyen, BS; Matthew Nguyen, MPH; Melissa Resendiz Rivas, BA; Melissa Boothe Scheer, PA-C; Janice Suyehira, MD; Stacie Wellwood, LPN; Maryann K Woodford, PA-C

**Department of Medicine, Division of Infectious Diseases and Global Public Health, University of California San Diego, La Jolla, CA**

Susan J. Little, MD; Thomas C.S. Martin, MD; Nicole Carter, MPH; Steven Hendrickx, RN; Ajay Bharti, MD; Alyssa Phillips; Aurora Verduzco Gonzalez, NP; Cheryl Dullano; Chris Houston; Dawn Rosenblum, RN; DeeDee Pacheco; DeLys Brooks; Fang Wan; Helene Le, CPhiT; JC Alcantar; Jill Blumenthal, MD; Joseph Lencioni, MABMH; Kory Hess; Letty Muttera, PharmD; Marlene Arredondo; Megan Smyth; Megan Taylor; Melinda Stafford, PharmD; Michelle Orsburn, MD; Michelle Truong; Niamh Higgins, PharmD, MSc, AAHIVP; Nimish Patel, PharmD, PhD, AAHIVP; Rebecca Gonzalez; Vivian Maldonado

**Morehouse School of Medicine, Atlanta, GA**

Lilly C. Immergluck, MD, MS; Erica Johnson, PhD; Austin Chan, MD; Fatima Ali, MPH; Sonja Jackson; Noor Mohamed, PharmD; LaKesha Tables, MD, MPH; Norberto Fas, MD; Kay Woodson, PharmD; Saadia Khizer, MD; Jacquelyn Ali, MSA; Abdullah Warsama; Eric Gaines; Sierra Jordan Thompson; Cristina Wilson; Trisha Parker, MPH; Xiting Lin; LaTeshia Thomas Seaton, APRN; Derrick Wilson

**Howard University College of Medicine, Howard University Hospital, Washington D.C.**

Siham M. Mahgoub, MD; Celia Maxwell, MD; Sarah Shami, PharmD; Edward Bauer, BS; Yuanxiu Chen, MD, PhD; Megan Ware-Pressley, MHA; Debra Ordor, RN; Linda Fletcher, RN; Emmanuel Baidoo, BS; David Jaspan, RPh, MBA; Adetokunbo Adedokun, PharmD, MPH, BCPS; Michelle Strobeck, BS; Michael A. Riga; Ashley Karen Bautista, BS

**Departments of Molecular Virology and Microbiology and Medicine, Baylor College of Medicine, Houston, TX**

Jennifer A. Whitaker, MD; Hana M. El Sahly, MD; Wendy A. Keitel, MD; C. Mary Healy, MD; Robert L. Atmar, MD; Pedro A. Piedra, MD; Jesus Banay; Kathy Bosworth; Janet Brown, RPh; Kayla Burrell; Jeremy Castro; Tykel Eddy; Marcena Eubanks; Cathy Faw, RPh; Rachel Froebe; Alix Halter, RN; Janey John, MSN, APRN, FNP-C; Chanei Henry, AAS; Vanessa Martinez; Carol Mundell, RN; Brandie Phillips, RN; Alicia Prevost-Barthe, RN; Connie Rangel, RN; Yolanda Rayford, MS; Yvette Rugeley; Maria Shlyapobersky; Tina Sierra; Elizabeth Silguero; Lisreina Toro; Dawn Turner, RN; Chianti Wade-Bowers, RN; Jessica Woods, RN; Robert L. Atmar, MD

**Departments of Medicine, Epidemiology, and Laboratory Medicine & Pathology, University of Washington, Vaccines and Infectious Diseases Division, Fred Hutchinson Cancer Center, Seattle, WA**

Tara M. Babu, MD, MSCI; Anna Wald, MD, MPH; Taylor Krause, BA; Kirsten Hauge, MPH; Jina Taub, ARNP; Dana Varon, ARNP; Britt Murphy, ARNP; Morissa Pertik, PA-C; T. Nui Pholsena, ARNP; Alyssa Braun, BS; ; Jessica Heimonen, MPH; Amy Link, BS; Lindsey McClellan, BS; Jessica Moreno, BS; Chloe Wilkens, BS; Matt Seymour, MPH; Lawrence Hemingway, BS; Jean Mernaugh, BS; Chris McClurkan, BS; Kerry Laing, PhD; Meredith Potochnic, PharmD; Joong Kim, PharmD; Bao-Chao Vo, PhT

**University of Alabama at Birmingham, Birmingham, AL**

Paul A. Goepfert, MD; Jenna Weber, RN; Savannah Spaulding, RN; Heather Logan, CRNP; Faye Heard; Foreamben Patel; Michelle Chambers

**Tulane University School of Medicine, New Orleans, LA**

Dahlene N. Fusco, MD; Arnaud C. Drouin, MD; Florice K. Numbi, MD; Hamada F. Rady, PhD; Crystal A. Ward, MSN; Quinn M. Powers, MS; William E. Casey, BS; Brian P. Logarbo, MD; Shae P. Williams, BS; Emily Callegari, MSN

**IDCRC Principal Investigators**

David S. Stephens, MD; Kathleen M. Neuzil, MD

**IDCRC Leadership Operations Center**

Monica M. Farley, MD; Jeanne Marrazzo, MD; Sidnee Paschal Young

**IDCRC Clinical Operations Unit**

Jeffery Lennox, MD; Robert L. Atmar, MD; Linda McNeil FHI360

**IDCRC Laboratory Operations Unit – Fred Hutchinson Cancer Center and University of Washington, Seattle, WA**

Christine M. Posavad, PhD; Megan A. Meagher, BS; Michael Stirewalt, MBA; John Hural, PhD; Weston Lawler, BA; Lexi Tanser, MA; Julie McElrath, MD, PhD; Mike Gale, PhD

**IDCRC Statistical and Data Science Unit**

Elizabeth Brown, PhD

**University of Maryland, Baltimore, MD**

Kirsten E. Lyke, MD

**FHI360, Durham, NC**

Kuleni Abebe, MSc

**The Emmes Company, LLC, Rockville, MD**

Mat Makowski, PhD; Heather Hill, MS; Jim Albert, MS; Holly Baughman; Lisa McQuarrie, MS; Kalyani Telu, MS; Jinjian Mu, PhD; Lisa J. McQuarrie, MSc

**Clinical Monitoring Research Program Directorate, Frederick National Laboratory for Cancer Research, Frederick, MD**

Teri C. Lewis, BS; Lisa A. Giebeig, MS; Theresa M. Engel, MFS.; Caleb J. Griffith, MPH; Wendi L. McDonald, BSN; Alissa E. Burkey, MS; Lisa B. Hoopengardner, MS; Jessica E. Linton, MS; Nikki L. Gettinger, MPH; Aroussiak Bowen; Beth R. Baseler, MS; Vanessa S. Eccard-Koons, MS; Charles W. R. Hofsommer, JD; Thomas C. Sova, JD; Gary A. Krauss

**Department of Surgery and Duke Human Vaccine Institute, Duke University School of Medicine, Durham, NC**

David C Montefiori, PhD; Amanda Eaton, MBA; Francesca Suman, MS.

**Smith’s Laboratory, Cambridge, UK**

Derek J Smith, PhD; Antonia Netzl; Samuel H Wilks, PhD; Sina Türeli, PhD; Ana Mosterín Höpping, PhD; Samuel Turner; Sarah James, MD; Poppy Roth

**Division of Microbiology and Infectious Diseases, National Institute of Allergy and Infectious Diseases, National Institutes of Health, Bethesda, MD**.

Marina Lee, PhD; Mamodikoe Makhene, MD; Mohamed Elsafy, MD; Rhonda Pikaart-Tautges, BS; Janice Arega, MS: Binh Hoang, RPh; Dan Curtin; Hyung Koo, BSN; Elisa Sindall, BSN; Aya Nakamura, RN, MS; Audria Crowder, BS; Guinevere Chun, RN, BSN, MSHS; Frank Kenny, PhD MPH; Seemi Patel, RHP, PharmD; Sonia Gales, MS; Ahsen Khan, JD; Walla Dempsey, PhD; Robert Jurao- RN, BSN; Sonja Crandon, BSN; Seema U. Nayak, MD; Marciela M DeGrace, PhD; Diane J Post, PhD; Paul C Roberts, PhD; John H Beigel, MD; SAVE Program

# Supplementary Methods

*Statistical methods*

Let *T* be the failure time from Day 15 (D15) nAb titer measurement until diagnosis of the COVID-19 primary endpoint between 7 days post D15 through 188 days post D15. Correlates of risk analyses are conducted separately for the Prototype and Omicron Vaccine Groups. We consider the following mark-specific proportional hazards (PH) model:

λ(*t*, *v*|*z*(*t*)) = λ_0_(*t*, *v*)exp { α_0_(*v*)BRS + α_1_(*v*)FOI + α_2_(*v*)NN + β_1_(*v*)nAb}

where BRS is the baseline risk score, FOI is the standardized FOI score, and NN is the indicator of being non-naïve, which is included in the analysis pooling over naïve and non-naïve. In addition, nAb is the D15 log10 nAb ID50 titer marker and Age is the participant’s age at enrolment.

*Implementation of the proposed estimation and testing procedures*

For imputation of COVID-19 primary endpoints with missing viral sequences, the nearest neighborhood *S_ki_* consists of the *z*-scores of *T_ki_* for participants with *δ_ki_* = 1, indicating observation of the dengue sequence distance. For each case *i* with missing dengue Hamming distance (i.e., “mark” *V_i_*), we used M = 5 imputed marks from the 5-nearest neighborhoods L_1_*_i_* calculated using *z*-scores of H_1_*_j_* = (*T*_1_*_j_*; *Age*_1_*_j_*; *nAb*_1_*_j_*) from all cases *j* with sequences observed.

*Choice of bandwidths for nonparametric kernel smoothing*

Viral sequence distance mark varoiables Marks were transformed using *V*^∗^ = *g*(*V*), where *g*(*v*) is the cdf of the normal distribution *N*(*µ*, σ^2^), where *µ* is the mean of the observed marks and σ is the standard deviation of the observed marks. Since histograms of the marks are mount-shaped and thin in the two tail areas, the benefit of such transformations is that we do not need to select different bandwidths for different marks. The estimator βˆ (*v*) of the v-specific log-hazard ratio coefficient function β(v) equals βˆ (*v*∗) through the inverse transformation *v*∗ = *g*^−1^(*v*).

We used the bandwidth *h* = 5σˆ*_v_n_o_*^-1/3^ as guidance for choosing the bandwidth, where σˆ *_v_* is the estimated standard error of the observed marks and *n_o_* is the number of COVID-19 endpoint cases. For the analysis of the Overall cohort, the standard deviation of the observed mark “*Hamming distances: nAb contact sites*” is 0.0225 for the vaccine group and 0.0243 for the placebo group, resulting in bandwidth *h* = 0.023 and *h* = 0.024, respectively. The bandwidth *h* for the transformed mark “*Hamming distances: nAb contact sites*”, is 0.024 for both the vaccine group and for the placebo group.

For the testing procedures for testing β_2_(*v*) = 0 and for testing β_2_(*v*) constant, we take *a* = min(*V*), *b* = max(*V*) and *a’* = *a* + 0.08.

*Hypothesis testing*

The null hypothesis H_10_ tested is whether D15 log10 nAb ID50 titer is correlated with COVID-19 for any specific distances of COVID-19 to a reference virus. The hypothesis is tested using the test statistic T_1a_ detailed in Sun et al. (2020), applied for the multiple analysis groups listed in Table 3 of the main article. The null hypothesis H_20_ tested is whether the distance-specific HR (per 10-fold increase in D15 log10 nAb ID50 titer) increases with distances of COVID-19 endpoints to a reference strain, again applied for the multiple analysis groups listed in Table 3 of the main article. The hypothesis is tested using the test statistic T_2m_ detailed in Sun et al. (2020).

**
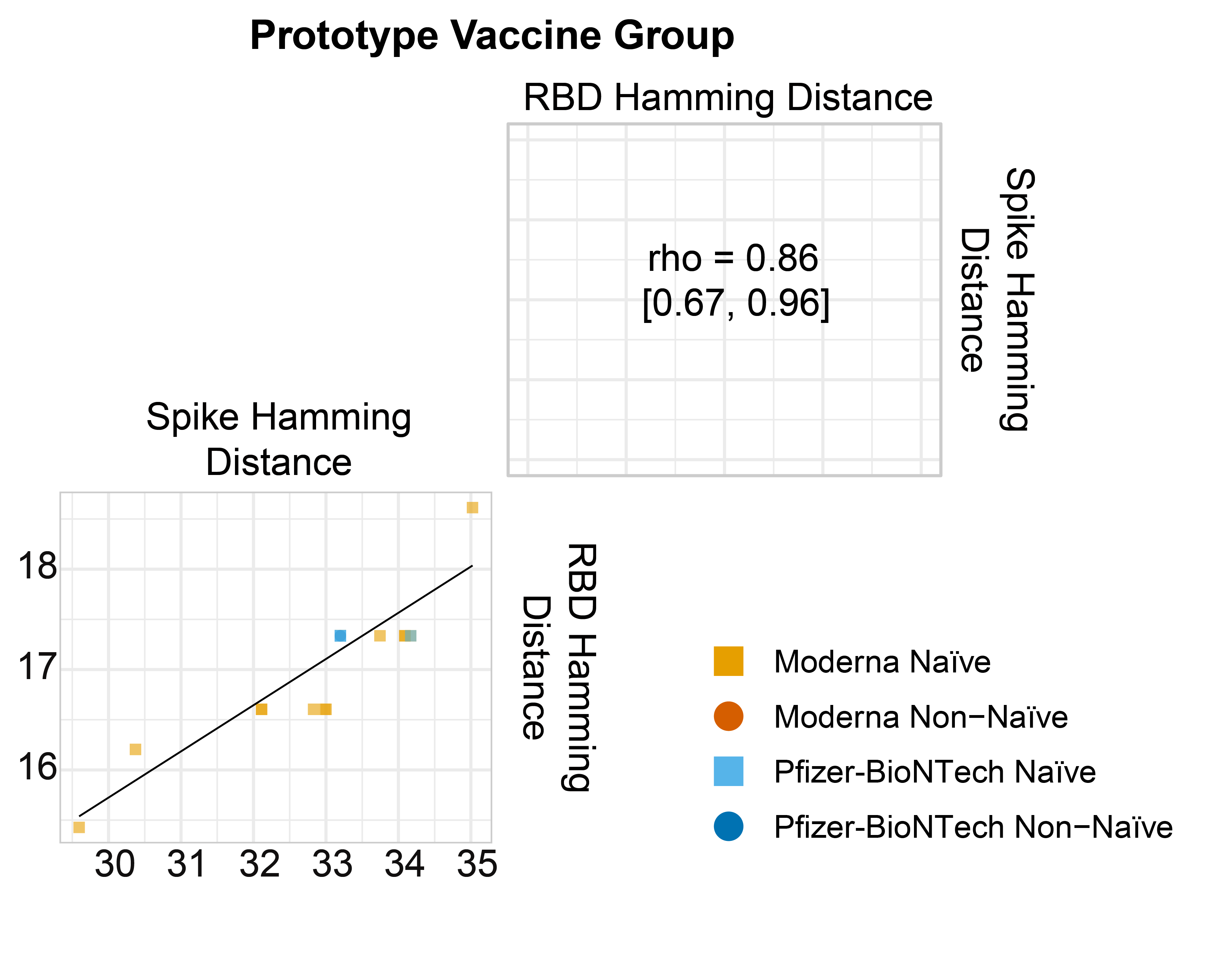
**

Supplementary Figure 1: Correlation of the Spike and RBD physico-chemical weighted Hamming distances for the Prototype Vaccine Group. Rho, Spearman rank correlation with a 95% confidence interval.

**

**

Supplementary Figure 2: Restricted to the Naïve cohort. For the viral sequence distances studied in immune correlates analyses, viral distances vs. Day 15 nAb ID50 titer for COVID-19 endpoint cases for the Prototype Vaccine Group and the Omicron Vaccine Group. For Prototype Vaccine Group arms, nAb ID50 titer is against D614G. For Omicron Vaccine Group arms, nAb ID50 titer is against BA.1 for all arms except Arm 17 for which it is against BA.4/5. Viral distances are physico-chemical weighted Hamming distances for Spike and RBD and three antibody escape score distances DMS-escape RBD-1, RBD-2, and RBD-3. Rho, Spearman rank correlation with a 95% confidence interval.

**
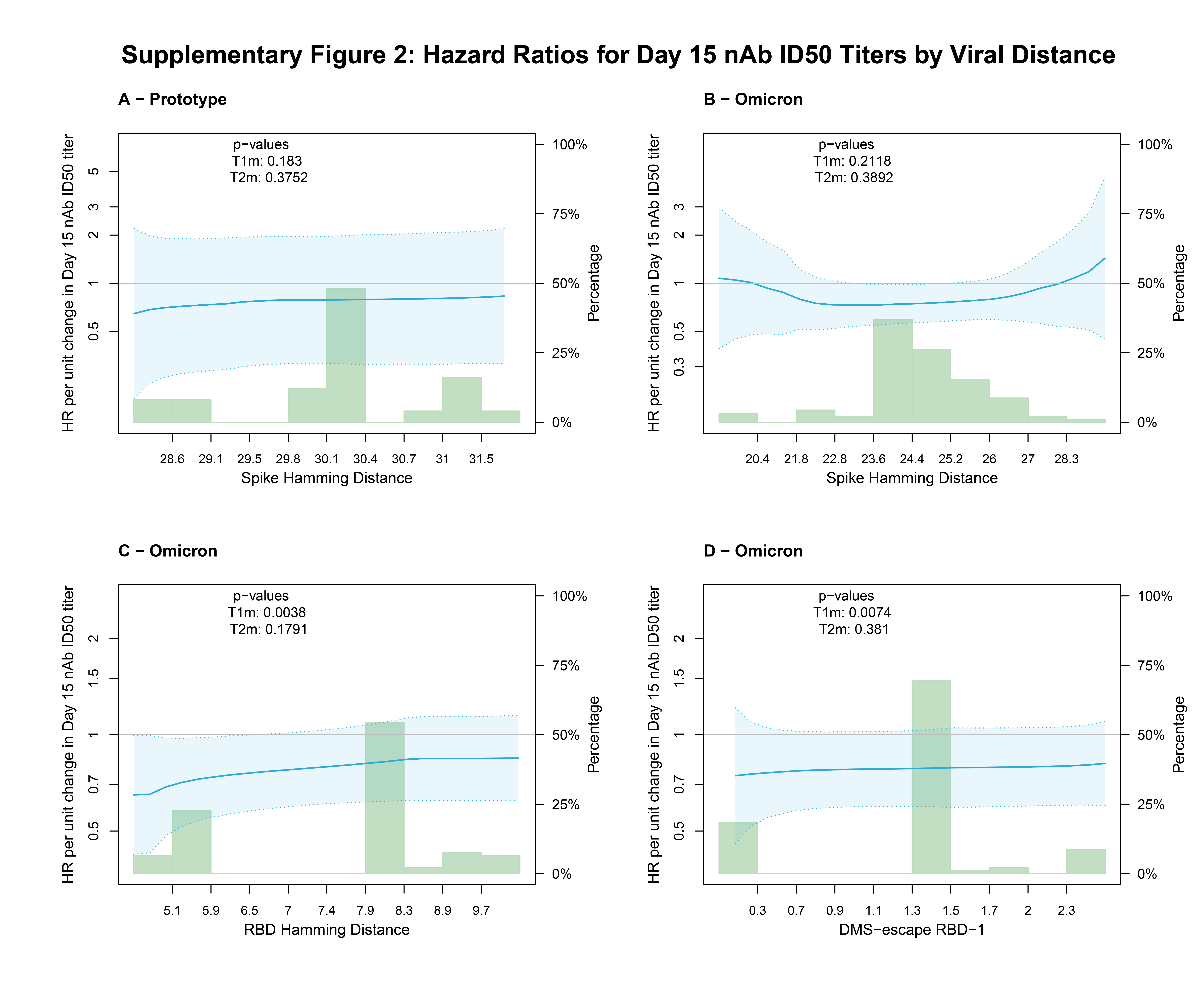
**

Supplementary Figure 3: Restricted to the Naïve cohort. Hazard ratios of viral distance-specific COVID-19 for Day 15 nAb ID50 titer markers for the Prototype Vaccine Group and the Omicron Vaccine Group. For Prototype Vaccine Group arms, nAb ID50 titer is against D614G. For Omicron Vaccine Group arms, nAb ID50 titer is against BA.1 for all arms except Arm 17 for which it is against BA.4/5. The 2-sided p-value for T1m tests whether nAb titer associates with COVID-19 for any distance and for T2m tests whether the association with COVID-19 varies with viral distance.

**
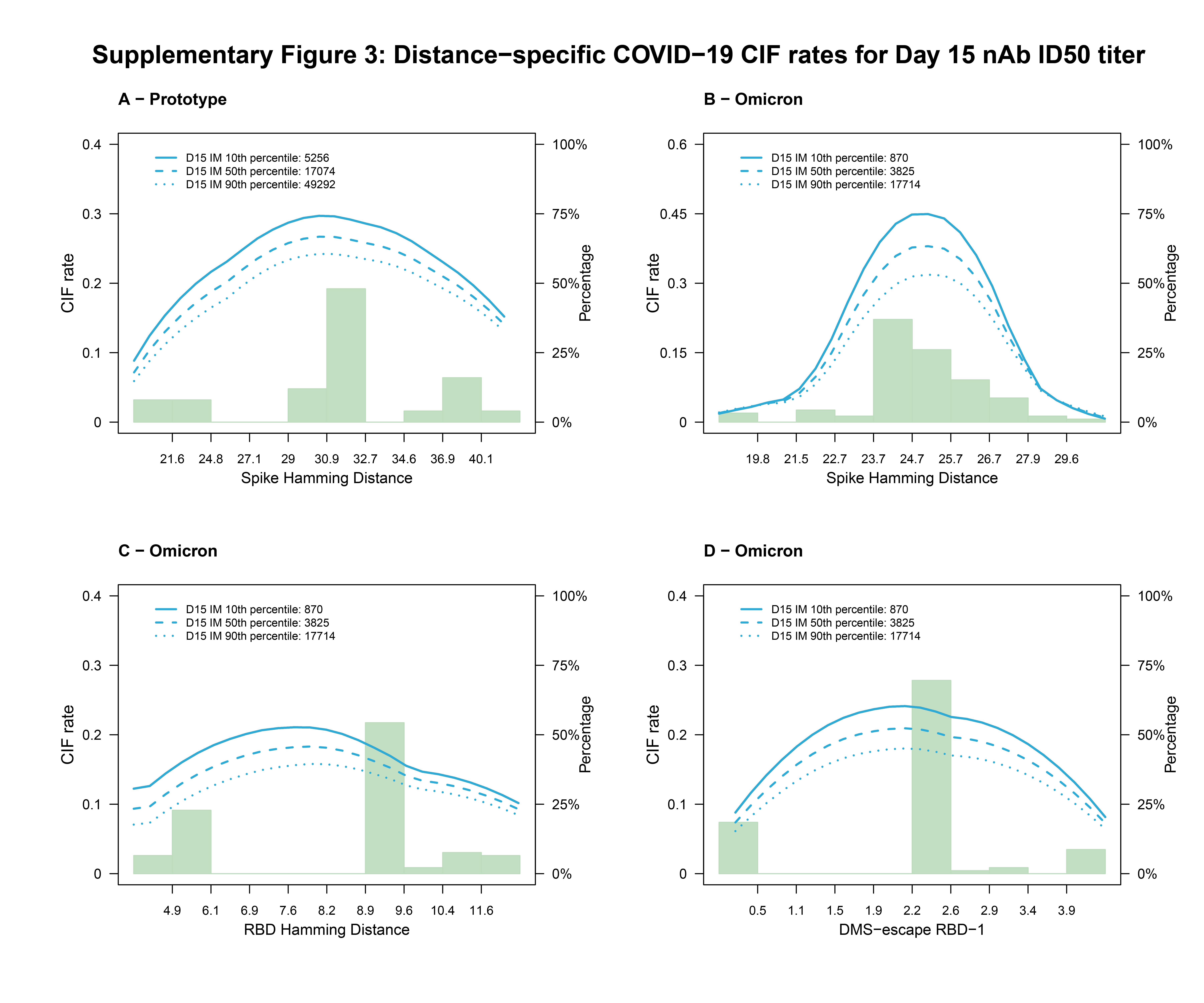
**

Supplementary Figure 4: Restricted to the Naïve cohort. Distance-specific COVID-19 Cumulative Incidence Function rates for Day 15 nAb ID50 titer markers set to the 10th, 50th, or 90th percentile values for the Prototype Vaccine Group and the Omicron Vaccine Group. For Prototype Vaccine Group arms, nAb ID50 titer is against D614G. For Omicron Vaccine Group arms, nAb ID50 titer is against BA.1 for all arms except Arm 17 for which it is against BA.4/5.
